# Knockdown of microRNA-214-3p Promotes Tumor Growth and Epithelial-Mesenchymal Transition in Prostate Cancer

**DOI:** 10.3390/cancers13235875

**Published:** 2021-11-23

**Authors:** Patrice Cagle, Nikia Smith, Timothy O. Adekoya, Yahui Li, Susy Kim, Leslimar Rios-Colon, Gagan Deep, Suryakant Niture, Christopher Albanese, Simeng Suy, Sean P. Collins, Deepak Kumar

**Affiliations:** 1 Julius L. Chambers Biomedical Biotechnology Research Institute, North Carolina Central University, Durham, NC 27707, USA; pcagle@nccu.edu (P.C.); nsmith47@nccu.edu (N.S.); tadekoya@nccu.edu (T.O.A.); yli6@nccu.edu (Y.L.); lrioscolon@nccu.edu (L.R.-C.); sniture@nccu.edu (S.N.); 2 Department of Cancer Biology, Wake Forest School of Medicine, Winston-Salem, NC 27157, USA; sukim@wakehealth.edu (S.K.); gdeep@wakehealth.edu (G.D.); 3 Wake Forest Baptist Comprehensive Cancer Center, Wake Forest School of Medicine, Winston-Salem, NC 27157, USA; 4 Lombardi Comprehensive Cancer Center, Department of Oncology, Georgetown University Medical Center, Washington, DC 20057, USA; Albanese@georgetown.edu; 5 Department of Radiation Medicine, Georgetown University Hospital, Washington, DC 20007, USA; suys@georgetown.edu (S.S.); spc9@georgetown.edu (S.P.C.); 6 Department of Pharmaceutical Sciences, North Carolina Central University, Durham, NC 27707, USA

**Keywords:** CRISPR/Cas9, microRNA, prostate cancer, EMT, RNA sequencing

## Abstract

**Simple Summary:**

Prostate Cancer is the second leading cause of cancer-related deaths in the United States. In this study, we analyzed a molecule known as a microRNA, which regulates the expression of genes. microRNAs are involved in processes related to cancer onset and progression. Abnormal expression of microRNAs can promote prostate cancer. This study showed that knockdown of microRNA miR-214-3p enhanced the progression and of prostate cancer. In addition, miR-214 regulated the expression of many genes. These results are useful to better understand the function of miR-214-3p in prostate cancer and can be a useful target in the treatment of the disease.

**Abstract:**

Abnormal expression of microRNA miR-214-3p (miR-214) is associated with multiple cancers. In this study, we assessed the effects of CRISPR/Cas9 mediated miR-214 depletion in prostate cancer (PCa) cells and the underlying mechanisms. Knockdown of miR-214 promoted PCa cell proliferation, invasion, migration, epithelial-mesenchymal transition (EMT), and increased resistance to anoikis, a key feature of PCa cells that undergo metastasis. The reintroduction of miR-214 in miR-214 knockdown cells reversed these effects and significantly suppressed cell proliferation, migration, and invasion. These in vitro studies are consistent with the role of miR-214 as a tumor suppressor. Moreover, miR-214 knockout increased tumor growth in PCa xenografts in nude mice supporting its anti-oncogenic role in PCa. Knockdown of miR-214 increased the expression of its target protein, Protein Tyrosine Kinase 6 (PTK6), a kinase shown to promote oncogenic signaling and tumorigenesis in PCa. In addition, miR-214 modulated EMT as exhibited by differential regulation of E-Cadherin, N-Cadherin, and Vimentin both in vitro and in vivo. RNA-seq analysis of miR-214 knockdown cells revealed altered gene expression related to PCa tumor growth pathways, including EMT and metastasis. Collectively, our findings reveal that miR-214 is a key regulator of PCa oncogenesis and is a potential novel therapeutic target for the treatment of the disease.

## 1. Introduction

Prostate cancer (PCa) is the most common cancer affecting men in the United States (U.S.) and is a second leading cause of cancer-related mortality in males [1]. According to the Surveillance, Epidemiology, and End Results (SEER) database, the 5-year survival rate for men diagnosed with prostate cancer in the U.S. is approximately 98% for localized PCa and 31% for metastatic PCa [2]. The current first-line treatments include radical prostatectomy and radiation treatment. Since PCa tumor growth is primarily driven by androgen receptor (AR) signaling, treatments include the use of drugs that decrease androgen synthesis or block binding of androgens to AR, called androgen deprivation therapy (ADT) [3,4]. These interventions typically show initial success, but virtually all patients eventually relapse with a more aggressive form of prostate cancer called castration-resistant prostate cancer (CRPC). Factors that influence progression of PCa to CRPC and metastatic disease include increased resistance to anoikis and increased EMT with its associated increase in invasive and metastatic properties [5]. The failure of existing therapies to slow the progression of metastatic PCa contributes to the majority of the deaths in PCa patients. Therefore, there is an urgent need to further understand molecular mechanisms underlying prostate cancer progression and metastasis and to identify novel therapies targeted to metastatic disease.

MicroRNAs (miRNAs) are a class of small noncoding RNAs (~22 nt) that are involved in the post-transcriptional regulation of genes by binding to the 3′ untranslated region (UTR) of target mRNAs and inhibiting translation or promoting their degradation. MiRNAs have been implicated in multiple physiological processes, including development, differentiation, growth, metabolism, cell signaling, and stress response [6,7,8]. Recent studies have shown that dysregulated miRNA expression is involved in cancer onset and progression, and miRNAs have been reported to regulate key oncogenic properties such as cell proliferation, apoptosis, angiogenesis, EMT, and tumor metastasis [9,10,11]. The functions of miRNAs are complex, and they can indeed have activities that promote tumor formation or block tumor formation.

MicroRNA-214-3p (miR-214) is derived from the miR-199a/miR-214 chromosomal locus 1q24.3 and its abnormal expression has been detected in several cancers, including hepatoblastoma, hepatocellular, gastric, esophageal, lung, breast, osteosarcoma, pancreatic, cervical, ovarian, bladder, oral, melanoma cancer, nasopharyngeal, and thyroid [12,13,14,15]. MiR-214 has been shown to play a critical role in cancer cell proliferation, cell cycle progression, apoptosis, migration, and invasion, as well as chemoresistance [16,17,18,19,20]. MiR-214 functions as a tumor suppressor by downregulating oncogenes such as FGFR1, HMGA1, and Hsp27 in colorectal cancer [17,19,21,22], SEMA4D in ovarian cancer [23], GALNT7, survivin, and CUG-BP1 in esophageal cancer [24,25], ARL2, Plexin-B1, and MKK3 in cervical cancer [26,27,28], and CRMP5 in PCa [29]. We and others have reported that miR-214 is downregulated in PCa tissue [16,29,30]. We further demonstrated that miR-214 targets PTK6, sensitizes PCa cells to Ibrutinib, and inhibits proliferation, migration, and invasion in PCa cells [16]. The objective of this study was to characterize the role of miR-214 in PCa growth and aggressiveness through the use of CRISPR-mediated knockout technology and RNA Sequencing (RNA-Seq) transcriptome analysis.

## 2. Materials and Methods

### 2.1. Cell Lines and Cell Culture

Parental (wild type miR-214–WT) and miR-214 knockout (KO) pooled prostate cancer cell lines, PC3 and MDA-PCa-2b, were constructed as described below. The PC3 miR-214KO D2 clonal cells are an independent clone isolated by single-cell dilution from a pool generated by target-specific sgRNA and Cas9. Cell lines were tested and authenticated using a short tandem-repeat analysis by LabCorp (Burlington, NC, USA). Cells were maintained in RPMI (PC3) and HPC1 medium (MDA-PCa-2b) supplemented with 5% or 10% fetal bovine serum respectively and 25 µg/mL gentamicin (Thermo Fisher Scientific, Waltham, MA, USA), and maintained in an incubator at 37 °C with 5% CO_2_. Clonal PC3 miR-214KO (D2 clone) cells were also used for several experiments and will be noted in the text.

### 2.2. Establishment of miR-214 Knockout Cells

The miR-214 single guide RNAs (sgRNAs) were designed, and the establishment of miR-214KO cells was generated by Synthego (Redwood City, CA, USA). The sgRNAs and SpCas9 protein were transfected into the cells via nucleofection as a ribonucleoprotein (RNP) complex, and miR-214 excision was confirmed by PCR and Sanger sequencing. The two sgRNA sequences were GGCCCCCGAGCCCCTCATT and ATCAGCCTGTTTTCCAGATT. The PCR and sequencing primer sequences for miR-214 were forward, 5′-TTTCCACCCTATCCCCTTCC-3′; reverse 5′-CTGACTACATGTGGGCCAGT-3′. The CRISPR/Cas9 miR-214 gene-edited PC3 and MDA-PCa-2b cells were electroporated with Cas9 and target-specific sgRNA and the parental WT isogenic negative control cells were electroporated with only Cas9 (no sgRNA).

PC3 and MDA-PCa-2b WT/miR-214KO cells were collected and whole genomic DNA was extracted by utilizing the Wizard(R) Genomic DNA Purification Kit (Promega, Madison, WI, USA). Platinum™ SuperFi™ PCR Master Mix was used to confirm the desired edit in cells. The amplifications were performed in an Applied Biosystems™ GeneAmp™ PCR System 2700 (Fisher Scientific) according to the manufacturer’s instructions. MiRNA expression was confirmed using quantitative RT-PCR (qRT-PCR). Clonal populations were also generated by Synthego for PC3 miR-214KO cells and the D2 clone was chosen for use in experiments, as noted.

### 2.3. miR-214 and Negative Control (NC) Mimic Transfection

Parental PC3 and MDA-PCa-2b cells (2–5 × 10^5^) were transiently transfected with 50 nM mirVana miR-214 mimic (Life Technologies Corporation, Carlsbad, CA, USA) or NC mimic (Life Technologies Corporation) using Lipofectamine 2000 reagent (Life Technologies Corporation) following the manufacturer’s protocol for 24 h. For miR-214 recovery assays, PC3 and MDA-PCa-2b miR-214KO cells were transiently transfected with 50 nM mirVana miR-214 mimic or NC mimic for 24 h.

### 2.4. MTT Assay

MTT assay was performed as previously described [16]. Briefly, PC3 and miR-214 knockout single-cell clones derived from PC3 cells were seeded in a 96-well plate at 5 × 10^3^ cells/well. Wild type and miR-214-knockout pooled MDA-PCa-2b cells were seeded at 6 × 10^3^ cells/well. After 24, 48, and 72 h, 5 mg/mL MTT was added to the medium, and the plate was incubated in a 37 °C, 5% CO_2_ incubator for 4 h. DMSO was added at a volume of 100 mL/well, and the plate was thoroughly mixed for 5 min. The absorbance of the samples was measured at 570 nm by a Fluostar Omega plate reader (BMG Lab Tech, Cary, NC, USA).

### 2.5. Clonogenic Assay

Colony formation was measured essentially as described by our group previously [16]. Parental wild-type or miR-214KO PCa cells were seeded at a density of 2000–5000 cells/well in a six-well plate and incubated at 37 °C with 5% CO_2_ for 7–10 days. The cells were then rinsed in PBS, fixed with 100% methanol, and stained with 0.5% crystal violet. The wells were photographed using the Bio-Rad Gel Doc XR and the number of colonies in the well was measured by ImageJ analysis (U.S. National Institutes of Health, Bethesda, MD, USA).

### 2.6. Anoikis Assay

Anoikis was measured with a CytoSelect 96-Well Anoikis Assay Kit (Cell Biolabs, Inc., San Diego, CA, USA) using MTT colorimetric and Calcein AM/EthD-1 fluorometric detection according to manufacturer’s instructions. Briefly, NC or miR-214 mimic transfected and wild-type or miR-214KO PCa cells (2 × 10^4^ cells/well) were seeded in a Poly-HEMA coated anchorage-resistant 96-well plate. After culturing for 48 h, MTT reagent was added to each well for 4 h at 37 °C, then detergent was added. The plate was incubated for another 4 h in the dark at room temperature, 150 μL was transferred to the 96-well plate, and absorbance was measured at 570 nm on the Fluostar Omega plate reader. Calcein AM (C-AM) and ethidium homodimer-1 (EthD-1) fluorescence was also detected following the manufacturer’s protocol, monitored under a Nikon TE-2000-E fluorescence microscope, and quantitatively measured with a Spectramax iD5 microplate reader (Molecular Devices) (C-AM, Ex: 485 nm and Em: 515 nm).

### 2.7. RNA Isolation and Quantification

Total RNA and microRNA from PC3 and MDA-PCa-2b WT and miR-214KO cells, as well as tissues, were isolated using the mirVana microRNA Isolation Kit (Thermo Fisher Scientific). Total microRNAs (10 ng) were reverse-transcribed using primers specific for miR-214 and U44 (Assay IDs 002306 and 001094, Applied Biosystems, Carlsbad, CA, USA) using Applied Biosystems™ PCR System 2720 (Thermo Fisher Scientific) and TaqMan Reverse Transcription reagents (Applied Biosystems). Expression of miR-214 and U44 was quantified by qRT-PCR using TaqMan PCR master mixture and TaqMan expression assay primers using Quant Studio 3 PCR System (Applied Biosystems), according to the manufacturer’s protocols.

For analysis of mRNA, total RNAs (1 µg) from PC3 and MDA-PCa-2b WT and miR-214KO cells were reverse transcribed using a High Capacity cDNA Reverse Transcription kit (Applied Biosystems). cDNA was incubated with Power SYBR Green PCR master mix (Applied Biosystems). Fold changes were determined by relative quantification (2^−ΔΔCt^ method). Primers for miRNA were ordered from Life Technologies and all primers for mRNA were synthesized by Integrated DNA Technologies (Coralville, IA), the sequences of all primers are shown in Table S1. U44 and GAPDH were used as the internal controls for miRNAs and mRNA, respectively.

### 2.8. Wound Healing Migration Assay

Wild type and miR-214KO PC3 cells were transfected with NC or miR-214 mimic and plated in a six-well plate overnight. Wound scratching assay, microscopy, and image analyses of PC3 cells were performed as previously described [16]. Briefly, two separate wounds were scratched using a 200 μL pipet tip, and cells were rinsed with cell culture medium. Pictures of the wound were taken at the same position under a Nikon TE-2000-E microscope at 0 h and 24 h. Migration ability was analyzed by quantifying the wound area using ImageJ as previously described [16].

### 2.9. Migration and Invasion Assay

Cell migration and invasion assays were performed using transwell chambers with 8 μm pore-size polycarbonate filters, noncoated (migration) or precoated with BME (invasion) according to manufacturer’s instructions. Cells were starved in serum-free media for 24 h and detached. For the invasion assay, the upper compartment was pre-coated with BME to form a matrix barrier in a 24-well plate. RPMI medium containing 10% FBS (0.5 mL) was added in the lower chamber to induce migration and invasion. Following 24 h (migration) or 48 h (invasion), the migrating/invading cells present on the lower surface of the filter member were stained with Calcein AM and incubated for 2 h at 37 °C in a cell culture incubator. Migrating/invading cell-associated fluorescence intensities were measured with a FluorStar Omega microplate reader using 485 nm excitation and 520 nm emission. The number of migrating/invading cells on the lower surface of the filter membrane was also observed and photographed using a Nikon TE-2000-E fluorescence microscope. The percent migration/invasion of miR-214KO cells was calculated using the formula (miR-214KO/WT) × 100. All analyses were performed in duplicate with three independent experiments.

### 2.10. Western Blot Analysis

Western blotting was performed as previously described [16]. Briefly, cell lysates were prepared from parental wild type or miR-214KO PC3 and MDA-PCa-2b cells using lysis buffer (Cell Signaling Technology, Danvers, MA, USA) containing a protease inhibitor cocktail (Roche, Indianapolis, IN, USA). Protein concentrations were determined using the Bio-Rad protein assay reagent (Bio-Rad, Hercules, CA, USA). Cell lysates (40 μg) were separated and transferred to a polyvinylidene difluoride membrane (Millipore, Billerica, MA, USA). Membranes were incubated overnight at 4 °C with anti-PCNA, VEGFR2, E-Cadherin, N-Cadherin, Vimentin, PTK6, and GAPDH (Cell Signaling Technology), CD31 (Life Technologies), VEGFA (Abcam), CXCR4, SESN3, PD-L1, ALK, and SAA1 (Abclonal Technology, and the appropriate HRP-conjugated secondary antibodies for 1 h at room temperature. All primary antibodies were used at a concentration of 1:1000. Following the incubation, membranes were washed, and protein bands were detected with Pierce ECL Western Blotting Substrate (Thermo Fisher Scientific, Waltham, MA, USA).

### 2.11. Immunofluorescence and Phalloidin Staining

For immunostaining, WT parental and miR-214KO PC3 cells (2.5 × 10^5^) were grown on coverslips in six-well plates. Cells were washed with PBS, fixed with 4% paraformaldehyde for 15 min, then cells were blocked with 2% bovine serum albumin for 1 h and incubated with anti-E-Cadherin (1:200), and anti-N-Cadherin (1:200) rabbit primary antibodies at 4 °C for 18 h. For Vimentin (1:200) staining, transfected cells were fixed and permeabilized with 0.3% Triton X-100 for 15 min and cells were blocked and incubated with anti-Vimentin rabbit primary antibody at 4 °C for 18 h. Cells were washed twice with PBS and incubated with Alexa Fluor^®^ 488-conjugated fluorescein-labeled (Invitrogen) for 2 h. The actin cytoskeleton was visualized using Alexa Fluor 594^®^ phalloidin (Life Technologies) for 30 min. Following immunostaining and washing, cells were mounted with ProLong™ Gold Antifade Mountant with DAPI (Life Technologies) for observation and viewed with a ZEISS LS 800 microscope.

### 2.12. RNA Isolation, Library Preparation, and RNA-Seq

MiRNA and total RNA were extracted from PC3 and MDA-PCa-2b wild type and miR-214KO PCa cells using a mirVana microRNA Isolation Kit as previously described [16]. RNA quantity was evaluated by NanoDrop 2000 (Life Technologies, Carlsbad, CA, USA). MiR-214 expression was validated before sequencing using qRT-PCR. Library preparation, RNA-Seq, and standard bioinformatics analyses were carried out by Genewiz, LLC (South Plainfield, NJ, USA). Heat map analysis and scatter plot analysis were conducted by applying the DEGseq2 R package to compare differentiated transcripts among samples based on log2 fold change > 1 and adjusted *p*-value < 0.05. Gene ontology analysis was carried out on the statistically significant set of genes using GeneSCF software. The goa human (gene ontology) GO list was used to cluster the set of genes based on their biological process and to determine their statistical significance.

### 2.13. In Vivo Tumorigenesis Assay

All animal experiments were carried out per the National Institutes of Health guide for the care and use of laboratory animals (NIH Publications No. 8023, revised 1978) and approved by the Institutional Animal Care and Use Committee. Six-week-old male athymic nude mice were purchased from Jackson Laboratories (Bar Harbor, ME, USA). To determine the effect of deletion of miR-214 on in vivo tumor growth, wild-type and miR-214KO PC3 cells (1.5 × 10^6^ cells) were injected subcutaneously into both flanks of 6-week of old athymic nude mice. Tumor size was assessed weekly and tumor volume was calculated as V = length × width^2^/2. Tumor weight was determined after euthanasia. Tumor sizes and weight were reported as mean volumes ± standard error of the mean (SEM). The mice were euthanized at 6 weeks, post-injection. Half of the tumor tissues were fixed in 10% formalin and embedded in paraffin, while the other half were harvested and stored at −80 °C for further studies. For RNA isolation, frozen tissue samples were soaked overnight in RNAlater^®^-ICE at −20 °C, transferred to a homogenizer containing lysis solution, and miRNA was isolated using mirVana microRNA Isolation Kit following the manufacturer’s protocol.

### 2.14. Immunohistochemical (IHC) Staining

IHC staining of tumor tissue was performed as previously described [31]. For IHC staining, deparaffinized sections were pretreated to retrieve antigens with a Tris-based Antigen Unmasking Solution (Vector Laboratories, Burlingame, CA, USA) before blocking with 10% normal serum and then applying either of the previously discussed antibodies and anti-PTK6 (Thermo Fisher Scientific) at 4 °C overnight. Tissue sections were then washed in PBS and incubated with biotinylated secondary antibodies for 30 min at room temperature. Detection of the antibody complex was carried out using the streptavidin–peroxidase reaction kit with DAB as a chromogen (ABC kit, Vector Laboratories).

### 2.15. Statistical Analysis

Data are expressed as mean ± SEM as previously described [16] of three independent experiments and each experiment was performed at least three times. Statistical significance between means was determined by Graph Pad Prism 9 software (GraphPad Software Inc., La Jolla, CA, USA) using one-way or two-way ANOVAs with Tukey’s multiple comparisons test (statistical significance determined using the Holm–Šídák method) when appropriate. Differences were considered significant at *p* < 0.05, and p-values are shown in the figures.

## 3. Results

### 3.1. Effects of miR-214 Gene Knockdown on Cell Proliferation and Colony Formation in Prostate Cancer Cells

**Figure 1:**
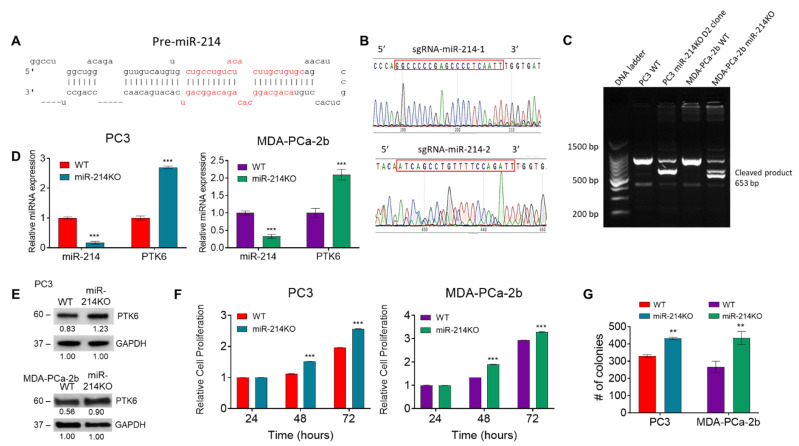
Downregulation of miR-214 in PCa cell lines using CRISPR/Cas9 leads to increased cell survival. (**A**) miR-214 pre-miRNA hairpin structure. (**B**) Two sgRNAs were designed for miR-214-3p depletion by using CRISPR DESIGN. (**C**) DNA cleavage by CRISPR/Cas9 is detected by PCR assay. (**D**) The relative expression of miR-214 and PTK6 was determined by qRT-PCR in parental (wild type-WT) PCa cells compared to miR-214KO PCa cells. MiRNA levels were normalized to that of U44 and Gapdh RNA. (**E**) Western blot analysis of PTK6 protein expression in PC3 and MDA-PCa-2b miR-214KO cells compared to WT cells. Original blots see Supplementary File S1. (**F**) Cell proliferation was evaluated using an MTT assay in PC3 and MDA-PCa-2b cells at 24 h, 48 h, and 72 h after seeding. (**G**) Cell colony formation assays showed the effect of knocking down miR-214 on PCa growth. The number of colonies was counted by ImageJ analysis (U.S. National Institutes of Health, Bethesda, MD, USA). Two-way ANOVA followed by Šídák’s multiple comparisons test was used to determine statistical significance. Data are presented as mean ± SEM, ** *p* < 0.005, *** *p* < 0.0005.

To further investigate the functional role of miR-214 in PCa, we performed knockdown studies using CRISPR/Cas9 gene-edited PC3 and MDA-PCa-2b cell lines. Proper insertion of sgRNAs by the introduction of a miR-214-directed Cas9/sgRNA complex was confirmed by PCR and Sanger sequencing. Two different pairs of guides targeting the complete miR-214 were selected (Figure 1A,B). The Platinum™ SuperFi™ PCR assay was used to recognize and cleave mismatched DNAs. As shown in Figure 1C, multiple site-specific bands were detected in PC3 miR-214KO D2 clone and MDA-PCa-2b miR-214KO groups compared to the WT groups, demonstrating the existence of miR-214 editing. CRISPR/Cas9 miR-214 gene editing was also confirmed in PC3 miR-214KO pooled cells using PC3 miR-214KO D2 clone cells as a positive control (Supplementary Figure S1A). After CRISPR/Cas9 targeting of miR-214 genomic sequences was performed in PC3 and MDA-PCa-2b cells, expression of miR-214 in the resulting miR-214KO cells was assessed by qRT-PCR. Our analysis showed that the expression of miR-214 was significantly decreased in both the PC3 miR-214KO D2 clonal cell line and MDA-PCa-2b miR-214KO cell line (Figure 1D). The knockdown was estimated to be 83% and 67% in PC3 and MDA-PCa-2b cell lines, respectively, showing the high efficiency of the CRISPR/Cas9 system. The relative expression of miR-214 was also determined in pooled miR-214KO PC3 cells compared to WT PC3 cells and a knockdown of 62% was observed (Supplementary Figure S1B). We’ve previously reported that the oncogene PTK6 is a direct target of miR-214 [16]. PTK6 transcript levels were considerably increased in PC3 D2 clonal miR-214KO cells and MDA-PCa-2b miR-214KO cells, consistent with decreased activity of miR-214 in these cells (Figure 1D). The protein expression of PTK6 was also increased under a similar miR-214KO condition in both PC3 miR-214KO pooled and MDA-PCa-2b cell lines (Figure 1E). The full length image of PTK6 western blot is shown in Supplementary File S1.E.

We next characterized the effect of miR-214 knockdown on cell proliferation. In the PC3 miR-214KO D2 clone cell line and MDA-PCa-2b miR-214KO line, the rate of cell proliferation was significantly increased at 48 and 72 h, compared with WT PC3 and MDA-PCa-2b cells (Figure 1F). In addition, clonogenicity was also significantly increased in PC3 (D2 clone) and MDA-PCa-2b miR-214KO cells compared to WT cells (Figure 1G), thus demonstrating an increased replicative ability in the absence of miR-214. Representative images of colonies are shown in Supplementary Figure S1C. These results indicate that genetic depletion of miR-214 promotes cell proliferation and colony formation of PCa cells.

### 3.2. miR-214 Knockdown Increases Anoikis Resistance, Invasiveness, and EMT in PCa Cells

**Figure 2:**
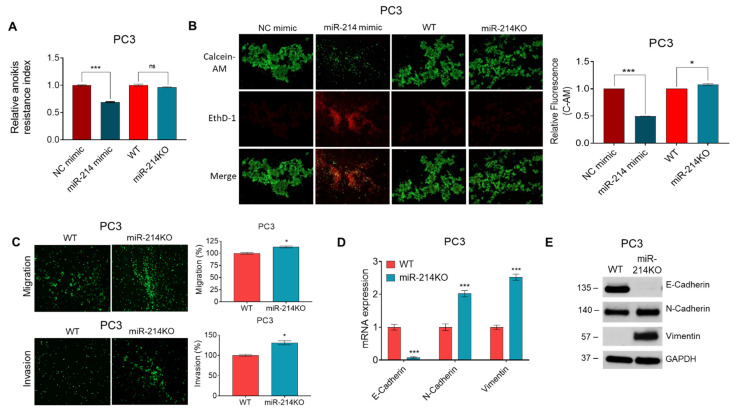
Inhibition of miR-214 expression increases PC3 cell anoikis resistance, migration, and invasion. PC3 cells transfected with miR-214 mimic or CRISPR/Cas9-deleted miR-214 were inoculated in a Poly-HEMA coated 24-well plate for 48 h. (**A**) Quantification of anoikis after 48 h using MTT assay of miR-214 mimic compared to NC mimic and miR-214KO normalized to WT PC3 cells. (**B**) Images and quantification of relative fluorescent staining of living cells (C-AM) and dead cells (EthD-1). (**C**) Transwell migration and invasion assays were used to determine the effect of miR-214 downregulation on PC3 cell migration and invasion (left panel). The percent of miR-214KO cells that migrated or invaded through the membrane was calculated with the formula (miR-214KO/WT) × 100 compared to WT (right panel). (**D**) Changes in E-Cadherin, N-Cadherin, and Vimentin mRNA in miR214-deleted PC3 cells normalized to its WT control were analyzed by qRT-PCR. (**E**) Western blot analysis revealed that knockdown of miR-214 significantly decreased E-Cadherin and increased N-Cadherin and Vimentin expression in PC3 cells. Original blots see Supplementary File S2. GAPDH was used as the loading control. One-way ANOVA and two-way ANOVA followed by Tukey’s and Šídák’s multiple comparisons test were used to determine statistical significance for anoikis and qRT-PCR assay, respectively. Data are presented as mean ± SEM, * *p* < 0.05, *** *p* < 0.0005, NS = not significant.

We have previously provided evidence for the role of miR-214 in promoting apoptosis in PCa cells [16]. However, whether miR-214 can influence anoikis has not been established. Anoikis is a specialized type of apoptotic cell death and plays an important role in tumor angiogenesis and metastasis [32,33]. Resistance to anoikis contributes to the capability of tumor cells to survive upon detachment from the extracellular matrix and dissemination from the primary tumor site into the systemic circulation, where they can successfully metastasize to distant organs [5,33]. We conducted our studies using miR-214 overexpressing PC3 cells as well as PC3 miR-214KO D2 clone cells and compared them to their respective controls (NC mimic and WT). To evaluate anoikis resistance, we measured the cell viability of suspended cells with an MTT assay. In these experiments, we observed that overexpression of miR-214 attenuated anoikis resistance of PC3 cells compared to cells transfected with NC mimic control at 48 h, while knockdown of miR-214 did not enhance anoikis resistance compared to WT cells (Figure 2A). We also performed C-AM/EthD-1 fluorometric detection using an anoikis assay. In the representative image at 48 h in Figure 2B (left panel), we observed an increase in EthD-1 (dead) cells and a decrease in C-AM (live) cells in miR-214 overexpressing PC3 cells compared to NC mimic. To further evaluate anoikis resistance, we quantified the living fluorescent cells and observed that the miR-214 mimic significantly reduced the viability of live nonadherent PC3 cells compared to NC mimic, while knockdown of miR-214 potentiated anchorage-independent growth compared to WT PC3 cells (Figure 2B, right panel). These results demonstrated that miR-214 expression could modulate anoikis resistance in PCa cells.

In our previous research, we demonstrated that miR-214 overexpression leads to reduced migration and invasiveness in PCa cells [16]. To determine whether these cell characteristics are affected by the knockdown of miR-214, we performed scratch and transwell assays in the PC3 miR-214KO D2 clone cell line. PC3 miR-214KO D2 clonal cells were found to have increased migration and invasion properties when compared to WT cells (Figure 2C). We next performed immunofluorescence assays with F-actin and anti-E-Cadherin, N-Cadherin, and Vimentin antibodies. We found that PC3 miR-214KO D2 clone cells had decreased expression of E-Cadherin and increased the expression of N-Cadherin and Vimentin compared to unmodified PC3 cells, but F-actin staining was not appreciably changed (Supplementary Figure S2A).

The expression of epithelial and mesenchymal genes and proteins as markers of EMT was examined in WT and miR-214KO D2 clone PC3 cells by qRT-PCR and Western blotting. As compared to the parental PC3 line, the epithelial marker E-Cadherin was significantly downregulated in miR-214KO D2 clone PC3 cells with a concomitant increase in expression in mesenchymal markers N-Cadherin and Vimentin (Figure 2D). Similarly, knockdown of miR-214 decreased E-Cadherin but increased N-Cadherin and Vimentin protein levels compared to parental PC3 cells (Figure 2E). The full length images of protein expression are shown in Supplementary File S2.E. Additional immunoblot analysis of PC3 miR-214KO D2 clone cell lysates support the significant decrease in E-Cadherin expression (Supplementary Figure S2B). Thus, knockdown of miR-214 enhanced markers of increased migration, invasion, and EMT in PC3 cells.

**Figure 3:**
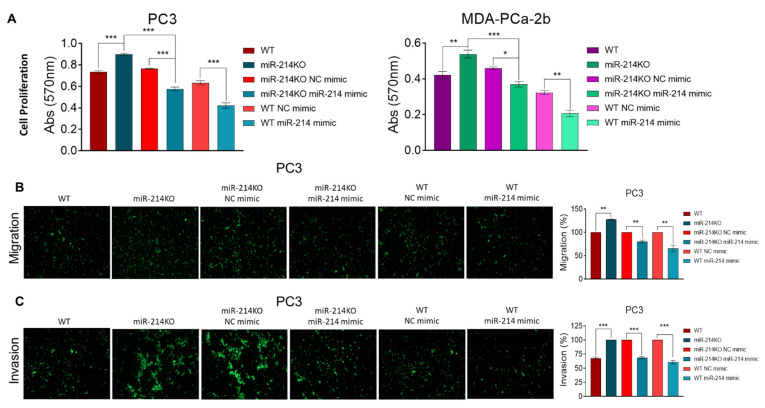
Overexpression of miR-214 partially reverses the silencing of miR-214 induced proliferative, migratory, and invasive effects. WT and miR-214KO PCa cells were transfected with NC mimic or miR-214 mimic. (**A**) After 48 h, the effect of the miR-214 expression on cell proliferation was determined by MTT assay. Migration (**B**) and invasion (**C**) ability were analyzed by transwell invasion assay in WT and miR-214KO miR-214 transfected PC3 cells, left panel, and quantified in the right panel. Pairwise normalization of WT vs miR-214KO; miR-214KO NC mimic vs miR-214KO miR-214 mimic; and WT NC mimic vs WT miR-214 mimic. Data are presented as mean ± SEM one-way ANOVA using Tukey’s multiple comparisons test, * *p* < 0.05, ** *p* < 0.005, *** *p* < 0.0005.

To provide more extensive verification of the function of miR-214, experiments were performed to determine whether miR-214 overexpression could reverse the effects of knockdown. Transfection of miR-214 mimic (versus NC mimic) was carried out in PC3 (miR-214KO pooled) and MDA-PCa-2b miR-214KO cells (Supplementary Figure S3A). Our data show that the level of miR-214 was markedly increased in the transfected cells, suggesting that we were able to restore the expression of miR-214 in miR-214-depleted cells. We’ve previously reported that overexpression of miR-214 attenuated cell proliferation, migration, and invasion of PCa cells [16]. In these experiments, the promotion of cell proliferation in PCa cells after miR-214 knockdown could be partially inhibited when the miR-214 levels were increased (Figure 3A). Furthermore, overexpression of miR-214 could partially abrogate the effects of downregulating miR-214 on PC3 cell migration (Supplementary Figure S3B; Figure 3B) and invasion ability (Figure 3C). Thus, we confirmed that overexpression of miR-214 could reverse the effects of the deletion of miR-214.

### 3.3. Differential Expression of mRNAs in PCa Cells with miR-214 Knockdown

**Figure 4:**
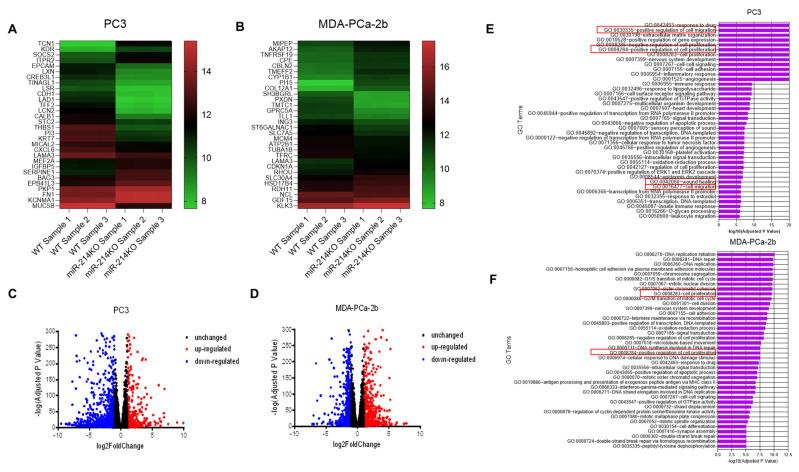
Identification and modulation of differentially expressed genes in miR-214-deleted PCa cells. Gene expression analysis was performed on the cells to compare the expression level of genes between WT and miR-214KO PCa cells. The differential miRNA expressional profiles from PC3 (**A**) and MDA-PCa-2b (**B**) cells miR-214KO compared with that in normal WT cells determined using RNA-Seq assays. Green represents relatively lower expression, and black/red indicates relatively higher expression. Volcano plot of the differentially expressed genes in PC3 (**C**) and MDA-PCa-2b (**D**) miR-214KO cells. Overexpressed genes are demonstrated in red and downregulated genes are demonstrated in blue. GO enrichment analysis of the differentially expressed mRNAs in PC3 (**E**) and MDA-PCa-2b (**F**) miR-214KO cells (Top 40 GO enrichment are presented, red box= enriched cell proliferation and migration processes).

To explore the mechanisms by which miR-214 mediates its effects in PCa, we assessed the changes in mRNA expression resulting from miR-214 knockdown in PCa cells, using RNA-Seq. Differential analysis of gene expression in the PC3 miR-214KO D2 clone and MDA-PCa-2b miR-214KO versus WT (parental) cell line control samples identified 1570 mRNAs (484 upregulated and 1086 downregulated) and 1774 (1008 upregulated and 766 downregulated) in PC3 and MDA-PCa-2b cells, respectively. A biclustering heatmap was used to visualize the expression profile of the top 30 differentially expressed genes (sorted by their adjusted *p*-value) by plotting their log2 transformed expression values in PC3 and MDA-PCa-2b samples (Figure 4A,B). The 50 most dysregulated mRNAs including the 25 most upregulated and 25 downregulated mRNAs in PC3 and MDA-PCa-2b miR-214KO cells are listed in Supplementary Tables S2 and S3. Differentially expressed RNAs in the dimensions of log2FC and –log (adj *p*-value) were visualized using a volcano plot (Figure 4C,D). We assessed the functional roles of the differentially expressed genes (DEGs) in PCa cells using GO enrichment analysis. The analysis revealed that numerous DEGs are involved in overlapping biological processes in both PC3 and MDA-PCa-2b cells, including cell proliferation, cell migration, angiogenesis, response to drug, nervous system development, cell adhesion, and cell–cell signaling (Figure 4E,F).

**Figure 5:**
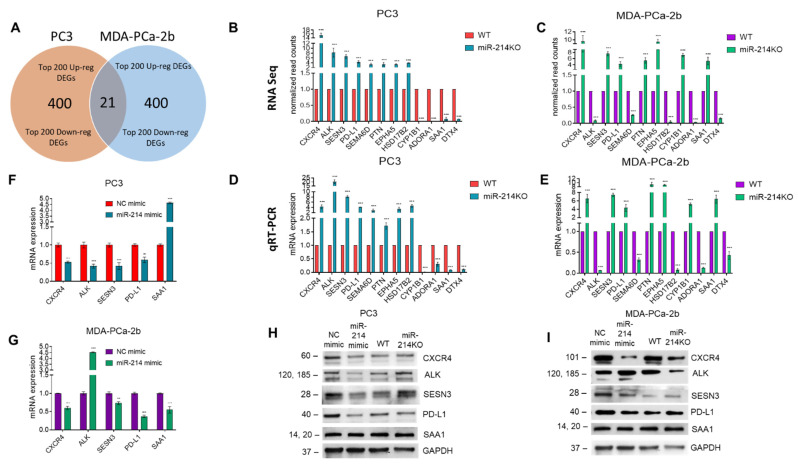
Modulation of overlapping target genes in miR-214-deleted PCa cells. (**A**) Venn diagram analysis of the top 400 aberrantly expressed mRNAs between WT and miR-214KO cells, as well as the overlapping genes between PC3 and MDA-PCa-2b groups. Normalized read counts of commonly regulated genes between PC3 (**B**) and MDA-PCa-2b (**C**) WT and miR-214KO groups. Relative expression levels of up/downregulated target genes were determined by qRT-PCR in PC3 (**D**) and MDA-PCa-2b (**E**) WT and miR-214KO cells. The mRNA (**F**,**G**) and protein expression (**H**,**I**) of CXCR4, ALK, SESN3, PD-L1, and SAA1 upon overexpression or knockdown of miR-214 was determined by qRT-PCR and Western blot analysis. Original blots see Supplementary File S5. Data are presented as mean ± SEM using two-way ANOVA with Šídák’s multiple comparisons test, ** *p* < 0.005, *** *p* < 0.0005.

A Venn diagram analysis was performed for the significant DEGs obtained from the top 200 up/downregulated genes from both PC3 and MDA-PCa-2b cells to identify the overlapping DEGs (Figure 5A). We found 21 genes that overlapped between the two cell lines. Of those 21 genes, eight genes were upregulated in both cell lines, four genes were downregulated, while the remaining nine genes were expressed in a cell line-specific manner. Importantly, 12 of the overlapping genes were previously validated miR-214-3p targets (CXCR4, SESN3, PD-L1, PTN, EPHA5, ADORA1, DTX4, ALK, HSD17B2, CYP1B1, SAA1, and SEMA6D). These genes were identified using our RNA-Seq analysis coupled with in silico tools, i.e., miRwalk, miRmap, Cytoscape/STRING, and Ingenuity Pathway Analysis. Further expression analysis was carried out on miR-214-3p target genes where an antibody was available for their protein. Using the normalized read counts from RNA-Seq, the expression of five genes (CXCR4, SESN3, PD-L1, PTN, and EPHA5) was upregulated while the expression of two genes (ADORA1 and DTX4) was downregulated in PC3 (Figure 5B) and MDA-PCa-2 cells (Figure 5C). The modulation of ALK, HSD17B2, CYP1B1, SAA1, and SEMA6D was cell-line-dependent. qRT-PCR was used to quantify the mRNA levels of the same 12 commonly regulated genes. We analyzed mRNA expression profiles in parental PC3 (Figure 5D) and MDA-PCa-2 (Figure 5E) cells versus knockdown cell lines using qRT-PCR and the results confirmed our observations from NGS. Conversely, overexpression of miR-214 significantly decreased CXCR4, SESN3, PD-L1 mRNA levels in both cell lines (Figure 5F,G), while expression of ALK and SAA1 was cell-line-dependent. The regulation of DEGs in miR-214 knockdown and overexpressing cells was further confirmed by analyzing the expression of CXCR4, SESN3, PD-L1 (PC3), as well as CXCR4 and PD-L1 (MDA-PCa-2b) proteins using a Western blot (Figure 5H,I). The full length protein expression images are shown in Supplementary Files S5.H and I.

### 3.4. Knockdown of miR-214 Enhanced PCa Tumor Growth, Angiogenesis, and EMT in Tumor Xenografts

**Figure 6:**
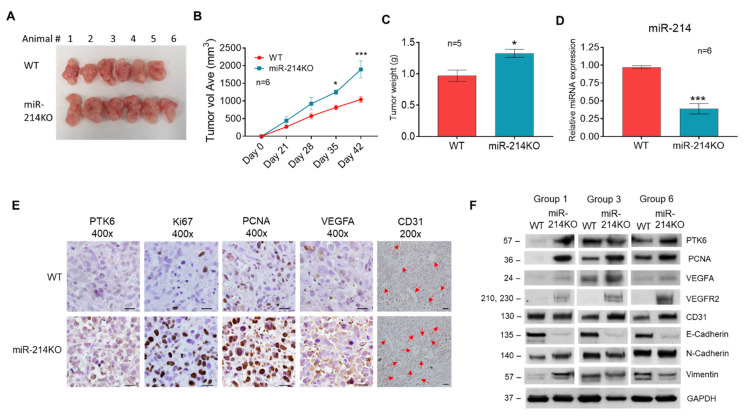
The effects of miR-214 on tumor growth, angiogenesis, and EMT in tumor xenografts. WT or miR-214KO PC3 cells were injected in nude male mice to form xenograft tumors. (**A**) Representative images of xenograft tumors at day 42 after injection. (**B**) Tumor volume in nude mice subcutaneously injected with miR-214 WT or KO PC3 cells. Data are presented as means ± SEM of the tumor volumes, calculated with the formula, V = length × width^2^/2. n = 6, * *p* < 0.05, *** *p* < 0.0005 by two-way ANOVA with Šídák’s multiple comparisons test between WT and KO groups. (**C**) Tumor growth of nude mice. (**D**) Quantification of expression of miR-214 by qRT-PCR as a percentage of U44 expression in micro-dissected xenograft prostate cancer cells. N = 6, *** *p* < 0.0005 (**E**) Representative IHC staining of hematoxylin and eosin, Ki67, PCNA, VEGFA, PTK6, and CD31 staining in xenograft prostate tumor tissues. Original magnification, ×400 (PTK6, Ki67, PCNA, VEGFA) and ×200 (CD31, red arrows = blood vessels). (**F**) Western blot expression of proliferative, angiogenesis, and EMT markers in PC3 WT and miR-214KO xenograft tumors. GAPDH was used as the loading control. Original blots see Supplementary File S6.

To add further evidence that miR-214 is involved in tumorigenesis, a xenograft model was developed whereby parental WT or miR-214KO D2 clone PC3 cells were subcutaneously injected into the left and right flank of immunodeficient nude male mice. After 6 weeks, xenograft tumor growth and weight were increased in mice that received the PC3 miR-214KO D2 clone cells compared with parental PC3 cells (Figure 6A–C). Reduced expression of miR-214 levels was confirmed in the PC3 miR-214KO tumors (Figure 6D). To analyze proliferation, angiogenesis, and EMT characteristics, tumor tissues were analyzed by immunohistochemical (IHC) staining with PTK6, Ki-67, PCNA, VEGFA, and CD31 antibodies. The positive rate of surface and subcellular markers Ki-67 and PCNA was significantly increased in the PC3 miR-214KO tumors compared to the control WT parental cell line-derived tumors, while the expression of VEGFA, CD31, and PTK6 in the PC3 miR-214KO group was slightly higher than that of the control group (Figure 6E). Western blot analysis also demonstrated similar results in expression patterns of PTK6, PCNA, VEGFA, VEGFR2, and CD31 in the tumors (Figure 6F). The full length protein expression images are shown in Supplementary Files S6.F and I. In agreement with what was found in the in vitro studies, protein levels of mesenchymal markers N-Cadherin and Vimentin were higher in PC3 miR-214KO tumors versus WT tumors, whereas protein levels of E-Cadherin were lower, which was consistent with increased EMT in PC3 prostate cancer cells. Taken together, these data indicate that the knockdown of miR-214 greatly enhanced the process of tumor progression in vivo and that miR-214 regulates tumorigenesis and metastasis via promoting proliferation, EMT, and VEGF-mediated angiogenesis.

## 4. Discussion

MiRNAs play a crucial function in diverse cellular processes by regulating gene expression through degradation and translation repression of target gene mRNAs [34]. Gain and loss of function approaches have been used to study the roles of miRNAs in cancer, both in vitro and in vivo. The CRISPR/Cas9 approach is one method that has been adopted for silencing miRNA expression by inserting mutations in the pre-miRNA sequences and disrupting miRNA expression [35,36,37]. Accumulating evidence in recent years has shown that abnormal expression of miRNAs is associated with oncogenesis, EMT, invasion, and metastasis [9,38].

Abnormal expression of miR-214 is found in various types of cancer. Evidence indicates that the expression of miR-214 is downregulated and acts as a tumor suppressor in multiple cancer types, including papillary thyroid carcinoma [14], hepatocellular carcinoma [39], esophageal [25], retinoblastoma [18], and prostate [16,29,30]. Recently, Zheng et al. [29] demonstrated that miR-214 expression is downregulated in human prostate cancer tissues when compared with normal tissues, which was consistent with our previous reports [16,30]. Moreover, using a gain of function approach, our research group previously showed that increased miR-214 expression inhibited cell clone formation, cell growth, migration, and invasion, and enhanced cell apoptosis [16]. Additionally, increased miR-214 expression sensitized PCa cells to Ibrutinib by modulating the activity of PTK6.

In the present study, we provide further insight into the role of miR-214 in PCa by disrupting its expression using CRISPR-mediated gene editing. This system allowed us to evaluate the effect of miR-214 knockdown on gene expression and gene regulatory networks in PC3 and MDA-PCa-2b cells by RNA Sequencing. Our study identified several key pathways/proteins regulated by miR-214.

One key tumor-related protein that was dysregulated by miR-214 gene editing was PTK6, a gene known to regulate oncogenesis in PCa cells. We previously showed that the 3′UTR of PTK6 is directly regulated by miR-214 and re-expression of PTK6 rescues PC3 and MDA-PCa-2b cell proliferation and clonogenicity. Our current data revealed that knockdown of miR-214 led to increased cell proliferation and clonogenic potential, and positively regulated PTK6 protein and mRNA expression in PC3 and MDA-PCa-2b cells. Moreover, this along with our previous studies, show that PTK6 expression positively correlates with PCa progression. Other studies have identified a link between PTK6, tumor cell survival, proliferation, cell cycle regulation, apoptosis, and metastasis [40]. PTK6 has been suggested to promote pancreatic cancer cell migration and invasion by activating ERK1/2 [41]. PTK6 has similarly been reported previously to regulate EMT and anoikis in PCa cells [42,43,44]. Indeed, the overexpression of PTK6 in prostate cancer cells promoted EMT via AKT activation, enhanced cell migration as well as metastasis in a prostate xenograft model [43]. In contrast, downregulation of PTK6 reversed EMT in triple-negative breast cancer cells and inhibited their growth and migration, but enhanced anoikis in vitro as well as suppressed metastatic ability in vivo through regulation of E-Cadherin [45]. Consistent with our results, miR-214 mimic inhibits proliferation, migration, and invasion of colon cancer, while downregulation promotes colon cancer growth and metastasis via regulation of PTK6 [46].

Tumor invasion and metastasis is a multistep process, which includes cancer cell growth, loss of cell adhesion, resistance to anoikis, and migration of cells away from the original tumor site to other parts of the body. The naturally occurring process of non-mobile epithelial cells into mobile, mesenchymal-like cells (also known as EMT) occurs during embryogenesis and tissue morphogenesis during development, as well as wound healing in the adult [47]. EMT results in the loss of cell polarity as well as the destruction of extracellular matrix and these have been observed during cancer progression and may lead to the development of metastatic tumor growth, drug resistance, and a poor prognosis [47,48]. In prostate cancer, data suggest that EMT contributes to PCa progression and metastasis and cells undergoing EMT acquire a migratory and invasive phenotype via resistance to anoikis, all of which contribute to therapeutic resistance and poor patient survival outcomes [48,49]. Progression to metastatic CRPC occurs in the stroma and is facilitated by angiogenesis, anoikis resistance survival mechanisms, and EMT [49,50].

Several miRNAs have been demonstrated to positively modulate anoikis resistance and anchorage-independent cell growth, including miR-133a-3p and miR23b/-27b. It was reported that upregulation of miR-133a-3p attenuates anoikis resistance in PCa cells, whereas silencing miR-133a-3p promotes anoikis resistance and prostate cancer bone metastasis via activating PI3K/AKT signaling [51]. Moreover, miR-23b/-27b suppressed CRPC cell migration, invasion, anchorage-independent growth, and tumor metastasis by downregulating Huntingtin interacting protein 1-related, while inhibition of miR23b/-27b in androgen-dependent prostate cancer cells increased migration and invasiveness [52,53]. Knockout of miR-210-3p by CRISPR-mediated silencing significantly increased renal cancer cell invasiveness in vitro and promoted tumorigenesis in xenograft experiments in vivo [54]. Downregulation of miR-338-3p promoted non-small-cell lung cancer (NSCLC) progression and metastasis in vitro and in vivo [55]. The results from our current study demonstrate that upregulating miR-214 increased anoikis in PC3 cells, while knockdown of miR-214 hindered this process.

Anchorage-independent cell growth can be suppressed by E-Cadherin, the loss of which is a characteristic feature of EMT [5]. Evidence was obtained during our study indicating that miR-214 deletion resulted in decreased E-Cadherin expression while enhancing N-Cadherin and Vimentin, providing further evidence that miR-214 modulates the EMT phenotype. In addition, knockout of miR-214 led to increased migration and invasion in PC3 cells and increased tumorigenicity and angiogenesis in xenograft models, thereby supporting a direct tumor suppressor role for miR-214 in preventing anchorage-independent cell growth and EMT. These data along with the anoikis results suggest that the promoting effect of miR-214 knockdown on PCa invasiveness and EMT might be partly attributed to anoikis resistance. Furthermore, this study showed that the effects of miR-214 knockdown on proliferation, migration, and invasion can be reversed by restoration of miR-214 in PCa cells, providing further support that miR-214 can play a role as a tumor suppressor to attenuate cancer progression.

Although we have previously shown that overexpression of miR-214 hinders PCa cell growth and migration/invasion by targeting PTK6 expression, the full complement of regulatory mechanisms underlying miR-214 effects in PCa is not completely known. To explore this further, RNA Sequencing was done using NGS technology. We observed that a total of 484 genes were upregulated and 1086 genes were downregulated between the PC3 samples, whereas a total of 1008 genes were upregulated and 766 genes were downregulated between the MDA-PCa-2b samples. Out of the total of 21 overlapping genes between the two cell lines, 12 genes showing significant changes were selected for further analysis (CXCR4, SESN3, PD-L1, PTN, EPHA5, ADORA1, DTX4, ALK, HSD17B2, CYP1B1, SAA1, and SEMA6D). The expression levels of each were validated by qRT-PCR. MiR-214 was found to downregulate CXCR4, SESN3, and PD-L1, while knockdown of miR-214 markedly increased CXCR4, SESN3, and PD-L1 above basal levels in PC3 and MDA-PCa-2b cells. The protein products of the genes showing regulation or downregulation in NGS showed similar trends in Western blotting experiments.

C-X-C motif chemokine receptor 4 (CXCR4), for example, has been reported as showing increased expression in several cancers, including endometrial cancer [56]. It is the most common chemokine receptor found in cancer cells and is associated with an aggressive disease phenotype, metastasis, and poor prognosis [57]. CXCR4 also enhanced proliferation and invasion in endometrial cells [56], while inhibition reduced cell proliferation and invasion, and induced cell cycle arrest in these cells [58]. In prostate cancer, increased CXCR4 expression was strongly associated with cell migration, metalloproteinase expression, invasion, and bone metastasis [4,59]. Indeed, the role of the CXCL12-CXCR4 signaling axis in PCa growth and metastasis has been extensively reviewed [60]. MiR-214 was found to downregulate CXCR4, while knockdown of miR-214 resulted in a fourfold and sevenfold increase of CXCR4 in PC3 and MDA-PCa-2b cells, respectively.

Anaplastic lymphoma kinase (ALK) is a receptor tyrosine kinase that belongs to the insulin receptor kinase subfamily. Activation or overexpression of ALK has been reported in many types of human cancer, including melanoma, NSCLC, neuroblastoma, glioblastoma, breast, colorectal, and thyroid. ALK activates multiple pathways (PI3K/AKT/mTOR, RAS/ERK, PLC-γ/DAG/PKC, and JAK/STAT), in which its downstream signaling leads to cell growth and survival, proliferation, transformation, angiogenesis, drug resistance, and metastasis [61,62,63]. ALK is activated in the metastatic CRPC lymph nodes, lung, liver, and bone lesions [64]. Moreover, ALK amplification and expression are significantly higher in CRPC with neuroendocrine differentiation (NEPC) patients compared to CRPC patients and are associated with poor survival outcomes [65].

Sestrin 3 (SESN3) is a stress response gene that protects the liver from carcinogen-induced hepatocellular carcinoma, and knockdown of SESN3 promotes carcinogen-induced hepatocellular carcinoma and metastasis by regulating hedgehog signaling, enhancing the production of extracellular matrix, and inducing cancer stem cell markers [66]. SESN3 is elevated in CRPC and the inhibition of SESN3 leads to cabazitaxel-induced ROS production [67]. In our studies, knockdown of miR-214 resulted in a significant increase of SESN3 in PC3 and MDA-PCa-2b cells, while miR-214 overexpression downregulated SESN3 levels. Since miR-214 is frequently downregulated in PCa tissue and cells, this could lead to higher levels of SESN3 and hence increased tumor growth and EMT in PCa.

Programmed cell death protein 1 ligand 1 (PD-L1), also known as cluster of differentiation 274 (CD274), is an immune system checkpoint regulator which has been implicated in various cancers. In claudin-low breast cancer cells, upregulation of PD-L1 expression is induced as part of the EMT process, whereas downregulation of PD-L1 is reversed in EMT [68]. In our studies, we observed that EMT and PD-L1 were significantly upregulated upon miR-214 knockout, while miR214 overexpression downregulated PD-L1 as well as reversed EMT (previously reported), suggesting possible crosstalk between EMT status and PD-L1 expression. Therefore, miR-214 negatively regulates PD-L1 levels and may play an important role in PD-L1 targeted therapy in prostate cancer. Serum amyloid A1 (SAA1) is an apolipoprotein secreted in response to inflammation and tissue injury and plays a vital role. Since there is a correlation between chronic inflammation and malignant transformation, studies suggest that SAA1 may affect oncogenesis and tumor metastasis [69]. Overexpression of SAA1 expression pancreatic cancer cells causes enhanced migration/ invasion capability, drug resistance, and EMT properties, while knockdown of SAA1 reverses these phenomena [70]. From our studies, we observed that SAA1 is highly expressed in MDA-PCa-2b cells (from AA origin) with knockdown of miR-214, while it is reduced in PC3 miR-214-deficient cells (from CA origin), suggesting it may behave differently in PCa depending on its origin.

## 5. Conclusions

In conclusion, our collective data revealed that the knockdown of miR-214 enhanced tumor cell growth, both in vitro and in vivo. The downregulation of miR-214 in PCa cells promoted cell proliferation, anchorage-independent growth, colony formation, migration/invasion, and EMT, consistent with the role of miR-214 as a tumor suppressor in PCa. These findings suggest that miR-214 may serve as a potential therapeutic target in PCa. Functional analyses indicated the existence of numerous genes whose expression is dysregulated in PCa cells by miR-214, in addition to PTK6, but additional studies are needed to clarify their roles in PCa. Our work also supports the need to carry out further research to delineate the role of miR-214 in advanced forms of disease such as CRPC, metastatic disease, and neuroendocrine PCa.

## Data Availability

Publicly available datasets were analyzed in this study. This data can be found here: Annotare 2.0 (E-MTAB-10782).

## Supplementary Materials

The following are available online at https://www.mdpi.com/article/10.3390/cancers13235875/s1, Figure S1: Downregulation of miR-214 in PCa cell lines, Figure S2: Immunofluorescence staining of EMT markers and E-Cadherin protein expression in PC3 cells, Figure S3: Effect of miR-214 overexpression in miR-214-depleted PCa cells, Table S1: Primer sets used for qRT-PCR, Table S2: Top 50 dysregulated mRNAs in PC3 cells, Table S3: Top 50 dysregulated mRNAs in MDA-PCa-2b cells, File S1(File S1.E\File S2.E\File S5.H\File S5.I\File S6.F\Supplementary Fig File S2.B): Original western blots.

## Abbreviations

Adenosine receptor A1, ADORA1; androgen deprivation therapy, ADT; Anaplastic lymphoma kinase, ALK; calcein acetoxymethyl ester/ethidium homodimer-1, Calcein AM/EthD-1; Castration-Resistant Prostate Cancer, CRPC; C-X-C motif chemokine receptor 4, Clustered Regularly Interspaced Short Palindromic Repeats/CRISPR-associated protein-9 nuclease, CRISPR/Cas9; CXCR4; Cytochrome P450 1B, CYP1B1; 4′,6-diamidino-2-phenylindole, DAPI; differentially expressed genes, DEGs; dimethylsulfoxide, DMSO; E3 ubiquitin-protein ligase, DTX4; enhanced chemiluminescence, ECL; Ephrin type-A receptor 5, EPHA5; knockout, KO; immunohistochemical, gene ontology, GO; Estradiol 17-beta-dehydrogenase 2, HSD17B2; IHC; microRNA-214-3p, miR-214; (3-(4,5-dimethylthiazol-2-yl)-2,5-diphenyltetrazolium bromide, MTT; Negative Control, NC; Next Generation Sequencing, NGS; non-small cell lung cancer, NSCLC; Proliferating cell nuclear antigen, PCNA; polymerase chain reaction, PCR; Programmed cell death protein 1 ligand 1, PD-L1; Pleiotrophin, PTN; prostate cancer, PCa; Protein Tyrosine Kinase 6, PTK6; quantitative real time PCR, qRT-PCR; Ribonucleoprotein, RNP; RNA Sequencing, RNA-Seq; Serum amyloid A1, SAA1; single guide RNAs, Semaphorin-6D, SEMA6D; Sestrin 3, SESN3; sgRNAs; standard error of mean (SEM); Surveillance, Epidemiology, and End Results, SEER; United States, U.S.; Vascular Endothelial Growth Factor A, VEGFA; Vascular endothelial growth factor receptor 2, VGFR2; wild type, WT; untranslated region, UTR.

## References

[1] Siegel R.L., Miller K.D., Fuchs H.E., Jemal A. (2021). Cancer Statistics, 2021. CA Cancer J. Clin..

[2] Howlader N., Noone A., Krapcho M., Miller D., Brest A., Yu M., Ruhl J., Tatalovich Z., Mariotto A., Lewis D. (2020). SEER Cancer Statistics Review, 1975–2017.

[3] Lin X., Kapoor A., Gu Y., Chow M.J., Xu H., Major P., Tang D. (2019). Assessment of biochemical recurrence of prostate cancer. Int. J. Oncol..

[4] Lee J.Y., Kang D.H., Chung D.Y., Kwon J.K., Lee H., Cho N.H., Choi Y.D., Hong S.J., Cho K.S. (2014). Meta-Analysis of the Relationship between CXCR4 Expression and Metastasis in Prostate Cancer. World J. Men’s Health.

[5] Cao Z., Livas T., Kyprianou N. (2016). Anoikis and EMT: Lethal “Liaisons” during Cancer Progression. Crit. Rev. Oncog..

[6] Ardekani A.M., Naeini M.M. (2010). The Role of MicroRNAs in Human Diseases. Avicenna J. Med. Biotechnol..

[7] Mendell J.T., Olson E.N. (2012). MicroRNAs in Stress Signaling and Human Disease. Cell.

[8] Chen B., Li H., Zeng X., Yang P., Liu X., Zhao X., Liang S. (2012). Roles of microRNA on cancer cell metabolism. J. Transl. Med..

[9] Peng Y., Croce C.M. (2016). The role of MicroRNAs in human cancer. Signal Transduct. Target. Ther..

[10] Ding X.-M. (2014). MicroRNAs: Regulators of cancer metastasis and epithelial-mesenchymal transition (EMT). Chin. J. Cancer.

[11] Tang J., Li Y., Wang J., Wen Z., Lai M., Zhang H. (2016). Molecular mechanisms of microRNAs in regulating epithelial-mesenchymal transitions in human cancers. Cancer Lett..

[12] Penna E., Orso F., Taverna D. (2015). miR-214 as a Key Hub that Controls Cancer Networks: Small Player, Multiple Functions. J. Investig. Dermatol..

[13] Sharma T., Hamilton R., Mandal C.C. (2015). miR-214: A potential biomarker and therapeutic for different cancers. Future Oncol..

[14] Liu F., Lou K., Zhao X., Zhang J., Chen W., Qian Y., Zhao Y., Zhu Y., Zhang Y. (2018). miR-214 regulates papillary thyroid carcinoma cell proliferation and metastasis by targeting PSMD10. Int. J. Mol. Med..

[15] Zhang H., Sun P., Wang Y.-L., Yu X.-F., Tong J.-J. (2020). MiR-214 promotes proliferation and inhibits apoptosis of oral cancer cells through MAPK/ERK signaling pathway. Eur. Rev. Med. Pharmacol. Sci..

[16] Cagle P., Niture S., Srivastava A., Ramalinga M., Aqeel R., Rios-Colon L., Chimeh U., Suy S., Collins S.P., Dahiya R. (2019). MicroRNA-214 targets PTK6 to inhibit tumorigenic potential and increase drug sensitivity of prostate cancer cells. Sci. Rep..

[17] Li W., Peng J., Chenhui Q., Xuefeng L., Lei W., Chunhong W. (2020). miR-214 sensitizes human colorectal cancer cells to doxorubicin by p53 targeting. Iran. Red. Crescent Med. J..

[18] Yang L., Zhang L., Lu L., Wang Y. (2020). miR-214-3p Regulates Multi-Drug Resistance and Apoptosis in Retinoblastoma Cells by Targeting ABCB1 and XIAP. OncoTargets Ther..

[19] Yang Y., Bao Y., Yang G.-K., Wan J., Du L.-J., Ma Z.-H. (2019). MiR-214 sensitizes human colon cancer cells to 5-FU by targeting Hsp27. Cell. Mol. Biol. Lett..

[20] Yu X., Luo A., Liu Y., Wang S., Li Y., Shi W., Liu Z., Qu X. (2015). MiR-214 increases the sensitivity of breast cancer cells to tamoxifen and fulvestrant through inhibition of autophagy. Mol. Cancer.

[21] Chen D.L., Wang Z.Q., Zeng Z.L., Wu W.J., Zhang D.S., Luo H.Y., Wang F., Qiu M.Z., Wang D.S., Ren C. (2014). Identification of MicroRNA-214 as a negative regulator of colorectal cancer liver metastasis by way of regulation of fibroblast growth factor receptor 1 expression. Hepatology.

[22] Chandrasekaran K.S., Sathyanarayanan A., Karunagaran D. (2016). MicroRNA-214 suppresses growth, migration and invasion through a novel target, high mobility group AT-hook 1, in human cervical and colorectal cancer cells. Br. J. Cancer.

[23] Liu Y., Zhou H., Ma L., Hou Y., Pan J., Sun C., Yang Y., Zhang J. (2016). MiR-214 suppressed ovarian cancer and negatively regulated semaphorin 4D. Tumor Biol..

[24] Lu Q., Xu L., Li C., Yuan Y., Huang S., Chen H. (2016). miR-214 inhibits invasion and migration via downregulating GALNT7 in esophageal squamous cell cancer. Tumor Biol..

[25] Phatak P., Byrnes K.A., Mansour D., Liu L., Cao S., Li R., Rao J.N., Turner D.J., Wang J.Y., Donahue J.M. (2016). Overexpression of miR-214-3p in esophageal squamous cancer cells enhances sensitivity to cisplatin by targeting survivin directly and indirectly through CUG-BP1. Oncogene.

[26] Peng R., Men J., Ma R., Wang Q., Wang Y., Sun Y., Ren J. (2017). miR-214 down-regulates ARL2 and suppresses growth and invasion of cervical cancer cells. Biochem. Biophys. Res. Commun..

[27] Qiang R., Wang F., Shi L.-Y., Liu M., Chen S., Wan H.-Y., Li Y.-X., Li X., Gao S.-Y., Sun B.-C. (2011). Plexin-B1 is a target of miR-214 in cervical cancer and promotes the growth and invasion of HeLa cells. Int. J. Biochem. Cell Biol..

[28] Peng R., Cheng X., Zhang Y., Lu X., Hu Z. (2020). miR-214 down-regulates MKK3 and suppresses malignant phenotypes of cervical cancer cells. Gene.

[29] Zheng C., Guo K., Chen B., Wen Y., Xu Y. (2019). miR-214-5p inhibits human prostate cancer proliferation and migration through regulating CRMP5. Cancer Biomark..

[30] Srivastava A., Goldberger H., Dimtchev A., Ramalinga M., Chijioke J., Marian C., Oermann E.K., Uhm S., Kim J.S., Chen L.N. (2013). MicroRNA Profiling in Prostate Cancer—The Diagnostic Potential of Urinary miR-205 and miR-214. PLoS ONE.

[31] Shi M., Ren S., Chen H., Li J., Huang C., Li Y., Han Y., Li Y., Sun Z., Chen X. (2021). Alcohol drinking inhibits NOTCH–PAX9 signaling in esophageal squamous epithelial cells. J. Pathol..

[32] Paoli P., Giannoni E., Chiarugi P. (2013). Anoikis molecular pathways and its role in cancer progression. Biochim. Biophys. Acta Mol. Cell Res..

[33] Strilic B., Offermanns S. (2017). Intravascular Survival and Extravasation of Tumor Cells. Cancer Cell.

[34] Bartel D.P. (2004). MicroRNAs: Genomics, biogenesis, mechanism, and function. Cell.

[35] Abdollah N.A., Kumitaa T.D., Narazah M.Y., Abdul Razak S.R. (2017). Sequence-specific inhibition of microRNA-130a gene by CRISPR/Cas9 system in breast cancer cell line. J. Phys.Conf. Ser..

[36] Zhao Y., Dai Z., Liang Y., Yin M., Ma K., He M., Ouyang H., Teng C.-B. (2014). Sequence-specific inhibition of microRNA via CRISPR/CRISPRi system. Sci. Rep..

[37] Cong L., Ran F.A., Cox D., Lin S., Barretto R., Habib N., Hsu P.D., Wu X., Jiang W., Marraffini L.A. (2013). Multiplex Genome Engineering Using CRISPR/Cas Systems. Science.

[38] Michael I.P., Saghafinia S., Hanahan D. (2019). A set of microRNAs coordinately controls tumorigenesis, invasion, and metastasis. Proc. Natl. Acad. Sci. USA.

[39] Xia H., Ooi L.L.P.J., Hui K.M. (2012). MiR-214 Targets β-Catenin Pathway to Suppress Invasion, Stem-Like Traits and Recurrence of Human Hepatocellular Carcinoma. PLoS ONE.

[40] Ostrander J.H., Daniel A.R., Lange C.A. (2010). Brk/PTK6 signaling in normal and cancer cell models. Curr. Opin. Pharmacol..

[41] Ono H., Basson M.D., Ito H. (2014). PTK6 promotes cancer migration and invasion in pancreatic cancer cells dependent on ERK signaling. PLoS ONE.

[42] Alwanian W.M., Tyner A.L. (2020). Protein tyrosine kinase 6 signaling in prostate cancer. Am. J. Clin. Exp. Urol..

[43] Zheng Y., Wang Z., Bie W., Brauer P.M., White B.E.P., Li J., Nogueira V., Raychaudhuri P., Hay N., Tonetti D.A. (2013). PTK6 activation at the membrane regulates epithelial-mesenchymal transition in prostate cancer. Cancer Res..

[44] Zheng Y., Gierut J., Wang Z., Miao J., Asara J.M., Tyner A.L. (2013). Protein tyrosine kinase 6 protects cells from anoikis by directly phosphorylating focal adhesion kinase and activating AKT. Oncogene.

[45] Ito K., Park S.H., Nayak A., Byerly J.H., Irie H.Y. (2016). PTK6 Inhibition Suppresses Metastases of Triple-Negative Breast Cancer via SNAIL-Dependent E-Cadherin Regulation. Cancer Res..

[46] Li T., Wan Y., Su Z., Li J., Han M., Zhou C. (2020). SRF Potentiates Colon Cancer Metastasis and Progression in a microRNA-214/PTK6-Dependent Manner. Cancer Manag. Res..

[47] Dongre A., Weinberg R.A. (2019). New insights into the mechanisms of epithelial–mesenchymal transition and implications for cancer. Nat. Rev. Mol. Cell Biol..

[48] Montanari M., Rossetti S., Cavaliere C., D’Aniello C., Malzone M.G., Vanacore D., Di Franco R., La Mantia E., Iovane G., Piscitelli R. (2017). Epithelial-mesenchymal transition in prostate cancer: An overview. Oncotarget.

[49] Wade C.A., Kyprianou N. (2018). Profiling Prostate Cancer Therapeutic Resistance. Int. J. Mol. Sci..

[50] Sakamoto S., Kyprianou N. (2010). Targeting anoikis resistance in prostate cancer metastasis. Mol. Aspects Med..

[51] Tang Y., Pan J., Huang S., Peng X., Zou X., Luo Y., Ren D., Zhang X., Li R., He P. (2018). Downregulation of miR-133a-3p promotes prostate cancer bone metastasis via activating PI3K/AKT signaling. J. Exp. Clin. Cancer Res..

[52] Ishteiwy R.A., Ward T.M., Dykxhoorn D.M., Burnstein K.L. (2012). The microRNA -23b/-27b cluster suppresses the metastatic phenotype of castration-resistant prostate cancer cells. PLoS ONE.

[53] Rice M.A., Ishteiwy R.A., Magani F., Udayakumar T., Reiner T., Yates T.J., Miller P., Perez-Stable C., Rai P., Verdun R. (2016). The microRNA-23b/-27b cluster suppresses prostate cancer metastasis via Huntingtin-interacting protein 1-related. Oncogene.

[54] Yoshino H., Yonemori M., Miyamoto K., Tatarano S., Kofuji S., Nohata N., Nakagawa M., Enokida H. (2017). microRNA-210-3p depletion by CRISPR/Cas9 promoted tumorigenesis through revival of TWIST1 in renal cell carcinoma. Oncotarget.

[55] Li Y., Chen P., Zu L., Liu B., Wang M., Zhou Q. (2016). MicroRNA-338-3p suppresses metastasis of lung cancer cells by targeting the EMT regulator Sox4. Am. J. Cancer Res..

[56] Liu P., Long P., Huang Y., Sun F., Wang Z. (2016). CXCL12/CXCR4 axis induces proliferation and invasion in human endometrial cancer. Am. J. Transl. Res..

[57] Zlotnik A. (2006). Chemokines and cancer. Int. J. Cancer.

[58] Long P., Sun F., Ma Y., Huang Y. (2016). Inhibition of CXCR4 and CXCR7 for reduction of cell proliferation and invasion in human endometrial cancer. Tumor Biol..

[59] Singh S., Singh U.P., Grizzle W.E., Lillard J.W. (2004). CXCL12–CXCR4 interactions modulate prostate cancer cell migration, metalloproteinase expression and invasion. Lab. Investig..

[60] Adekoya T.O., Richardson R.M. (2020). Cytokines and Chemokines as Mediators of Prostate Cancer Metastasis. Int. J. Mol. Sci..

[61] Hallberg B., Palmer R.H. (2013). Mechanistic insight into ALK receptor tyrosine kinase in human cancer biology. Nat. Rev. Cancer.

[62] Holla V.R., Elamin Y.Y., Bailey A.M., Johnson A.M., Litzenburger B.C., Khotskaya Y.B., Sanchez N.S., Zeng J., Shufean M.A., Shaw K.R. (2017). ALK: A tyrosine kinase target for cancer therapy. Cold Spring Harb. Mol. Case Stud..

[63] Kong X., Pan P., Sun H., Xia H., Wang X., Li Y., Hou T. (2019). Drug Discovery Targeting Anaplastic Lymphoma Kinase (ALK). J. Med. Chem..

[64] Drake J.M., Graham N.A., Lee J.K., Stoyanova T., Faltermeier C.M., Sud S., Titz B., Huang J., Pienta K.J., Graeber T.G. (2013). Metastatic castration-resistant prostate cancer reveals intrapatient similarity and interpatient heterogeneity of therapeutic kinase targets. Proc. Natl. Acad. Sci. USA.

[65] Carneiro B.A., Pamarthy S., Shah A.N., Sagar V., Unno K., Han H., Yang X.J., Costa R.B., Nagy R.J., Lanman R.B. (2018). Anaplastic Lymphoma Kinase Mutation (ALK F1174C) in Small Cell Carcinoma of the Prostate and Molecular Response to Alectinib. Clin. Cancer Res..

[66] Liu Y., Kim H.G., Dong E., Dong C., Huang M., Liu Y., Liangpunsakul S., Dong X.C. (2019). Sesn3 deficiency promotes carcinogen-induced hepatocellular carcinoma via regulation of the hedgehog pathway. Biochim. Biophys. Acta Mol. Basis. Dis..

[67] Kosaka T., Hongo H., Miyazaki Y., Nishimoto K., Miyajima A., Oya M. (2017). Reactive oxygen species induction by cabazitaxel through inhibiting Sestrin-3 in castration resistant prostate cancer. Oncotarget.

[68] Alsuliman A., Colak D., Al-Harazi O., Fitwi H., Tulbah A., Al-Tweigeri T., Al-Alwan M., Ghebeh H. (2015). Bidirectional crosstalk between PD-L1 expression and epithelial to mesenchymal transition: Significance in claudin-low breast cancer cells. Mol. Cancer.

[69] Sun L., Ye R.D. (2016). Serum amyloid A1: Structure, function and gene polymorphism. Gene.

[70] Takehara M., Sato Y., Kimura T., Noda K., Miyamoto H., Fujino Y., Miyoshi J., Nakamura F., Wada H., Bando Y. (2020). Cancer-associated adipocytes promote pancreatic cancer progression through SAA1 expression. Cancer Sci..

