# Supplementary material for: Knockdown of microRNA-214-3p Promotes Tumor Growth and Epithelial-Mesenchymal Transition in Prostate Cancer
Source: Cancers (Basel). 2021 Nov 23;13(23):5875. doi: 10.3390/cancers13235875 (PMC8656576; doi:10.3390/cancers13235875)
Supplement: Supplementary file 1 [file cancers-13-05875-s001.zip › cancers-1450969-supplementary/Original Western blots Final v2 11-22-21.pdf]

Knockdown of microRNA-214-3p Promotes Tumor Growth and Epithelial-Mesenchymal Transition in Prostate Cancer

Patrice Cagle [1], Nikia Smith [1], Timothy O. Adekoya [1], Yahui Li [1], Susy Kim [2], Leslimar Rios-Colon [1], Gagan Deep [2,3], Suryakant Niture [1], Christopher Albanese [4], Simeng Suy [5], Sean P. Collins [5] and Deepak Kumar [1,6,*]

[1] Julius L. Chambers Biomedical Biotechnology Research Institute, North Carolina Central University, Durham, NC 27707, USA; pcagle@nccu.edu (P.C.); nsmith47@nccu.edu (N.S.); tadekoya@nccu.edu (T.O.A.); yli6@nccu.edu (Y.L.); lrioscolon@nccu.edu (L.R-C.); sniture@nccu.edu (S.N.)
[2] Department of Cancer Biology, Wake Forest School of Medicine, Winston-Salem, NC 27157, USA; sukim@wakehealth.edu (S.K.); gdeep@wakehealth.edu (G.D.)
[3] Wake Forest Baptist Comprehensive Cancer Center, Wake Forest School of Medicine, Winston-Salem,
NC 27157, USA
[4] Lombardi Comprehensive Cancer Center, Department of Oncology,
Georgetown University Medical Center, Washington, DC 20057, USA; Albanese@georgetown.edu
[5] Department of Radiation Medicine, Georgetown University Hospital, Washington, DC 20007, USA; suys@georgetown.edu (S.S.); spc9@georgetown.edu (S.P.C.)
[6] Department of Pharmaceutical Sciences, North Carolina Central University, Durham, NC 27707, USA
*Correspondence: dkumar@nccu.edu; Tel.: +1-919-530-7017

File S1.E

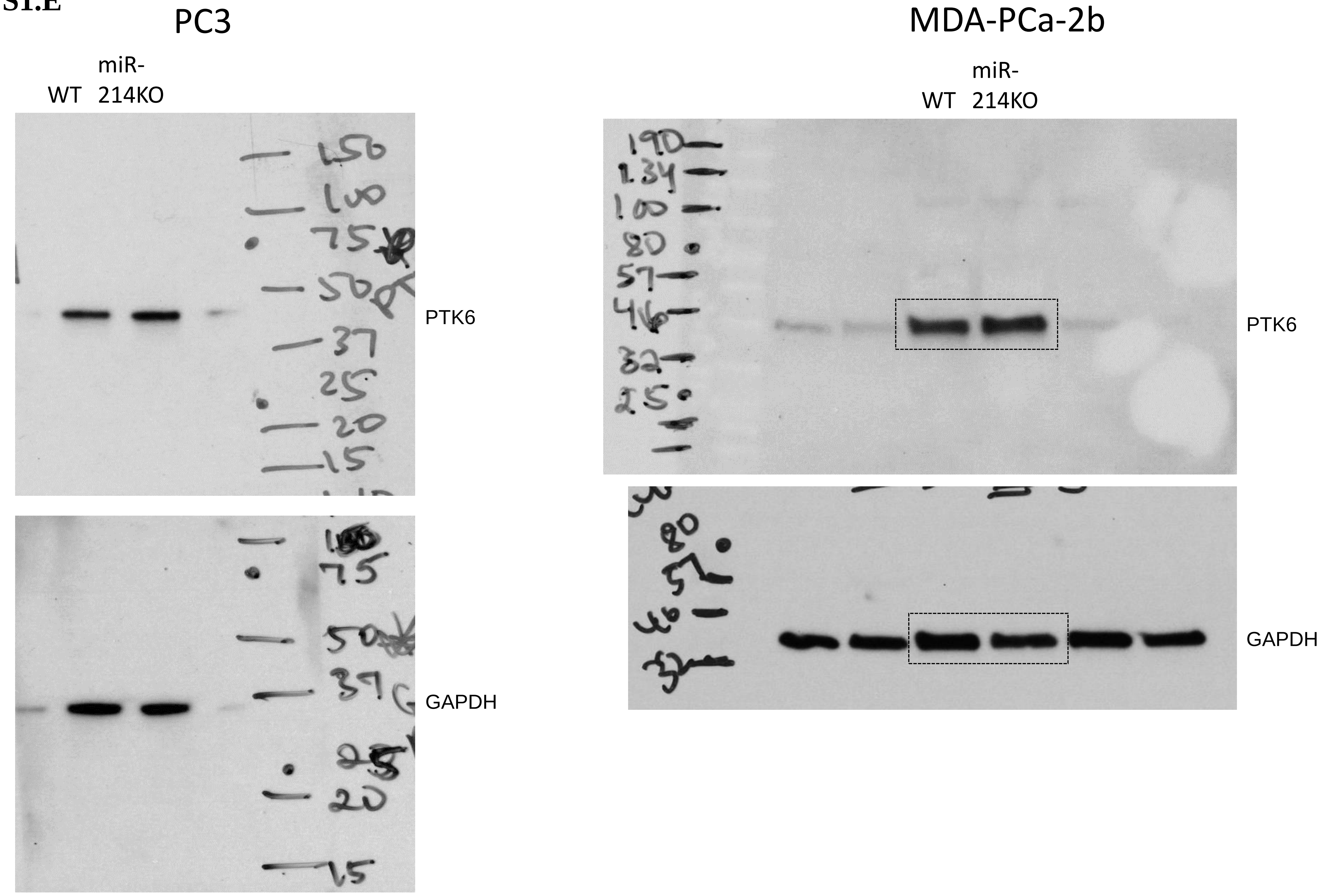

File S1.E (S1.E): Full length images of blots for Fig 1 E in main paper. Dotted rectangles indicate the regions used in the figures. PVDF membranes were used for probing with the respective primary antibody.

File S2.E

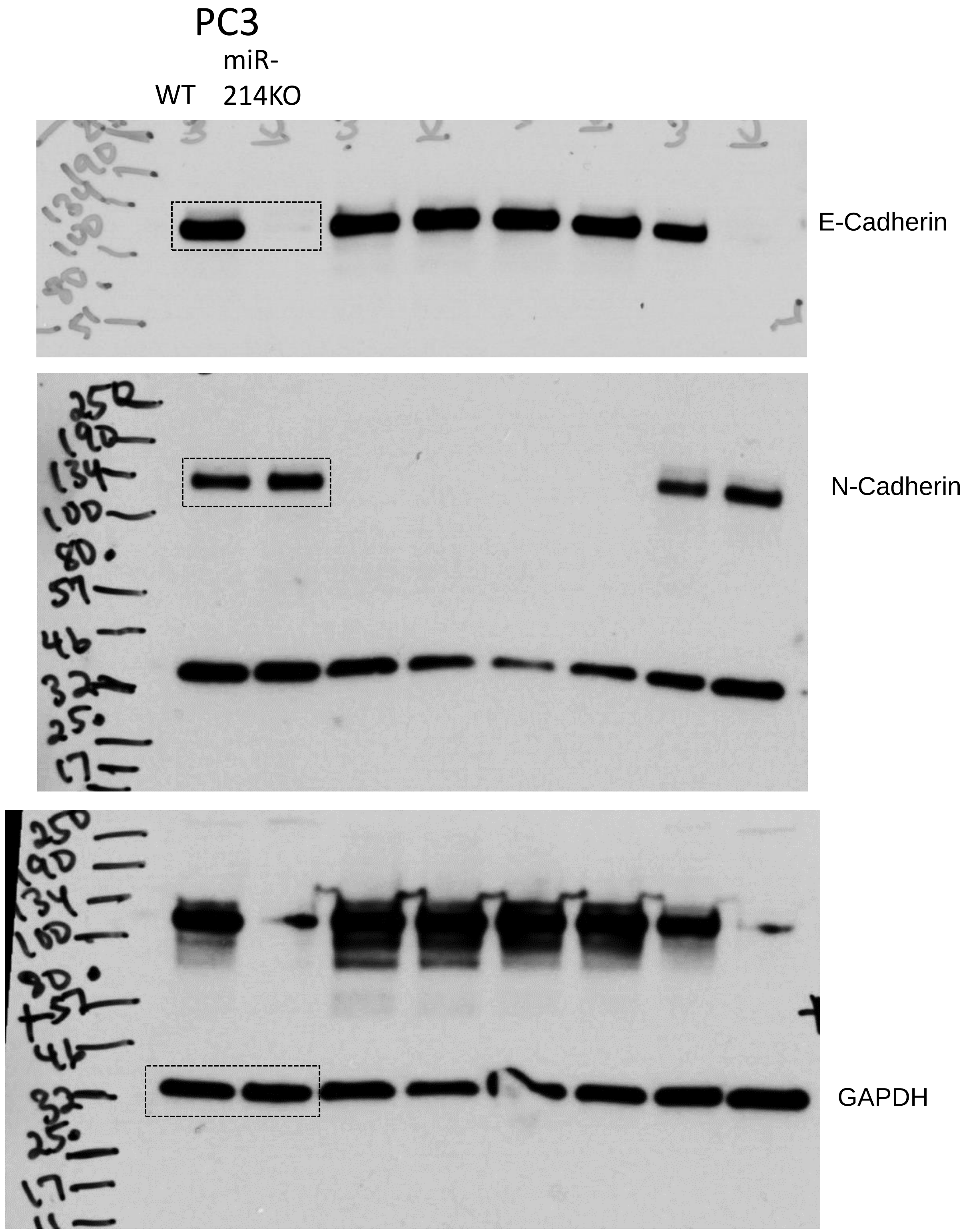

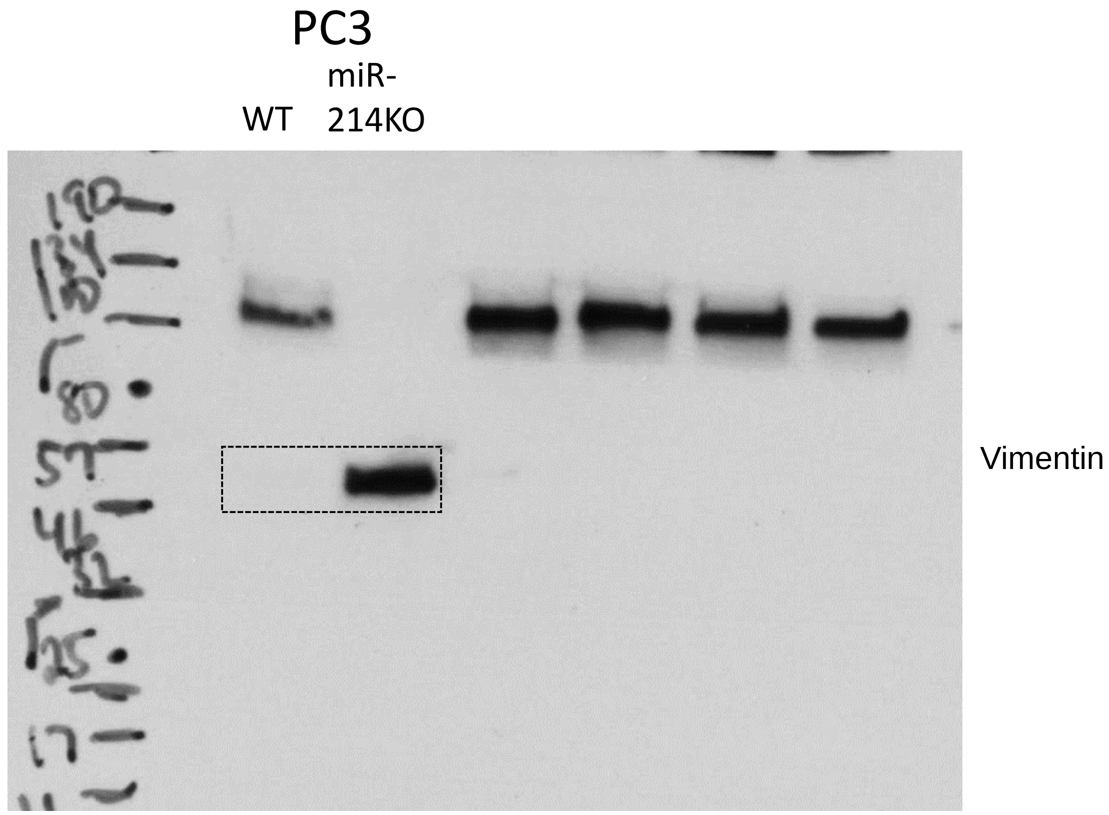

File S2.E (S2.E): Full length images of blots for Fig 2 E in main paper. Dotted rectangles indicate the regions used in the figures. PVDF membranes were used for probing with the respective primary antibody.

File S5.H

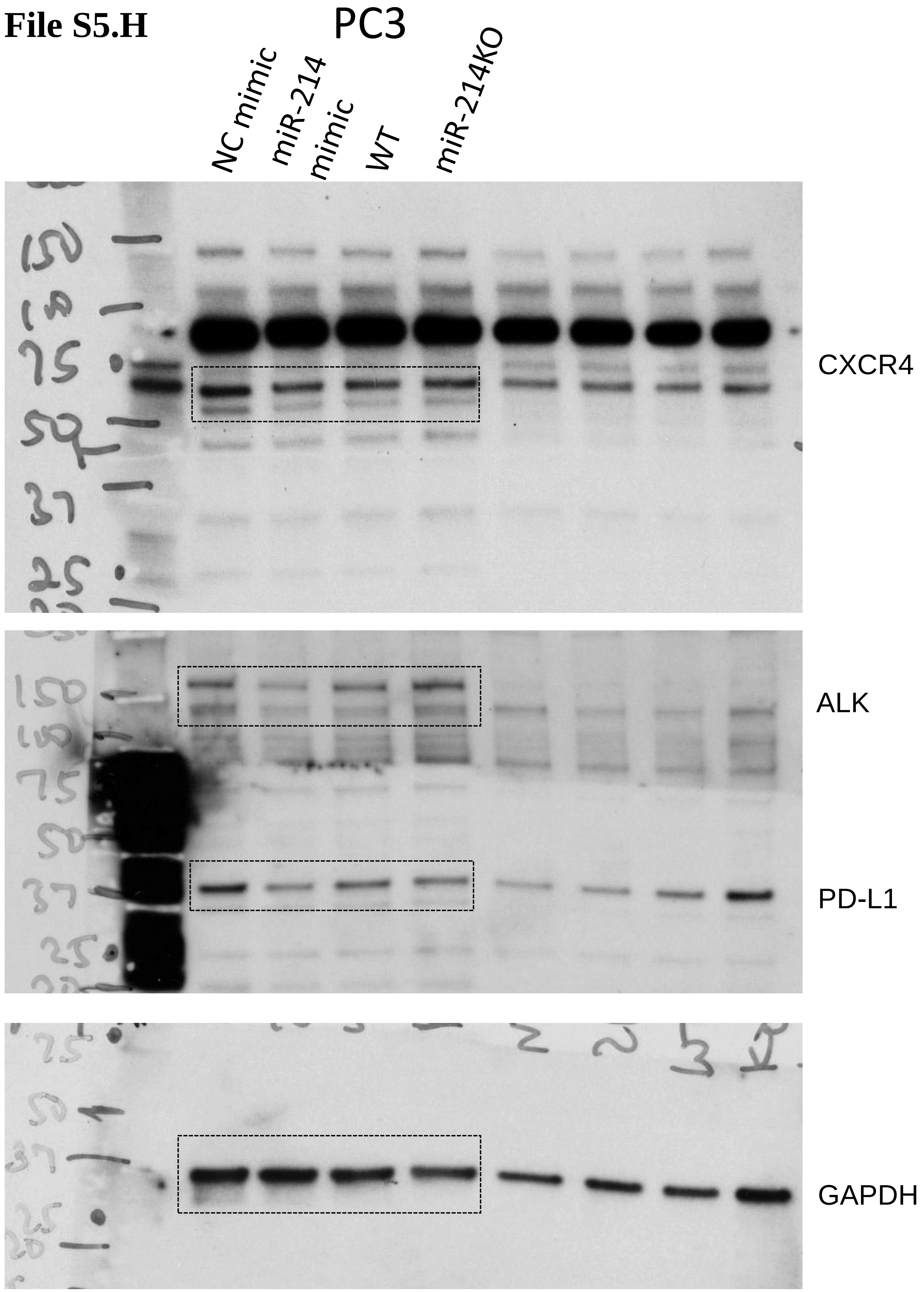

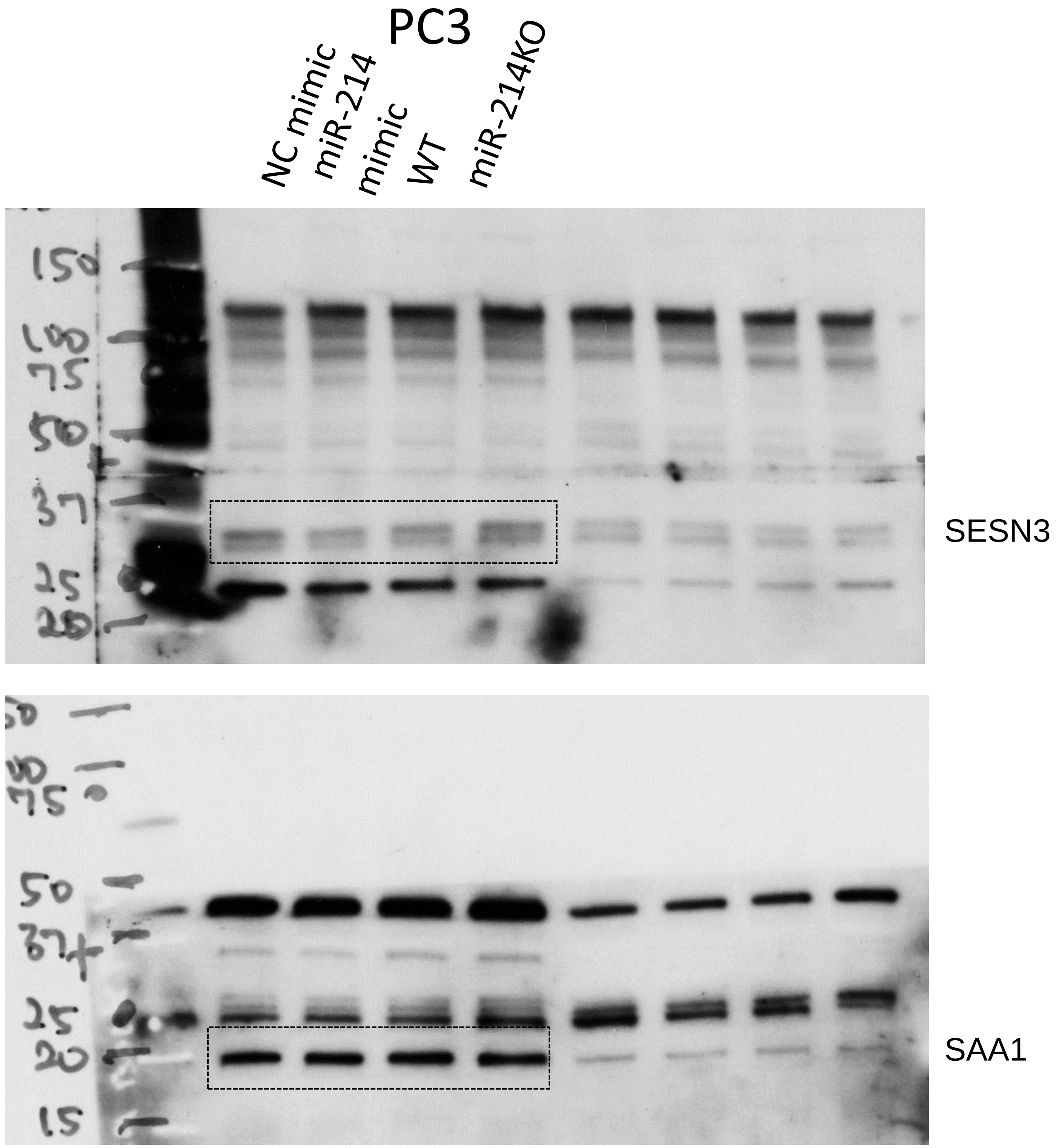

File S5.H (S5.H): Full length images of blots for Fig 5 H in main paper. Dotted rectangles indicate the regions used in the figures. PVDF membranes were used for probing with the respective primary antibody.

File S5.I

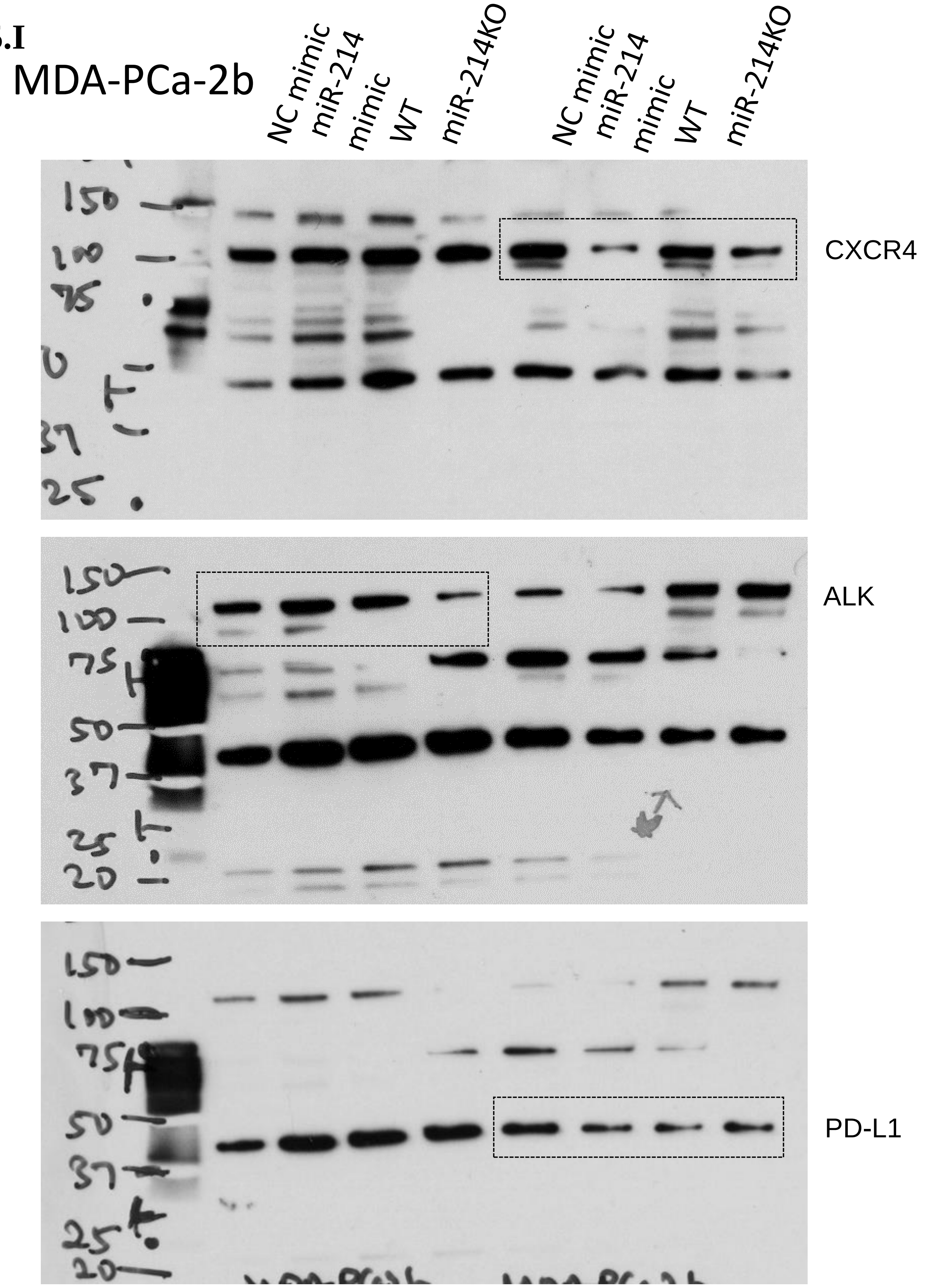

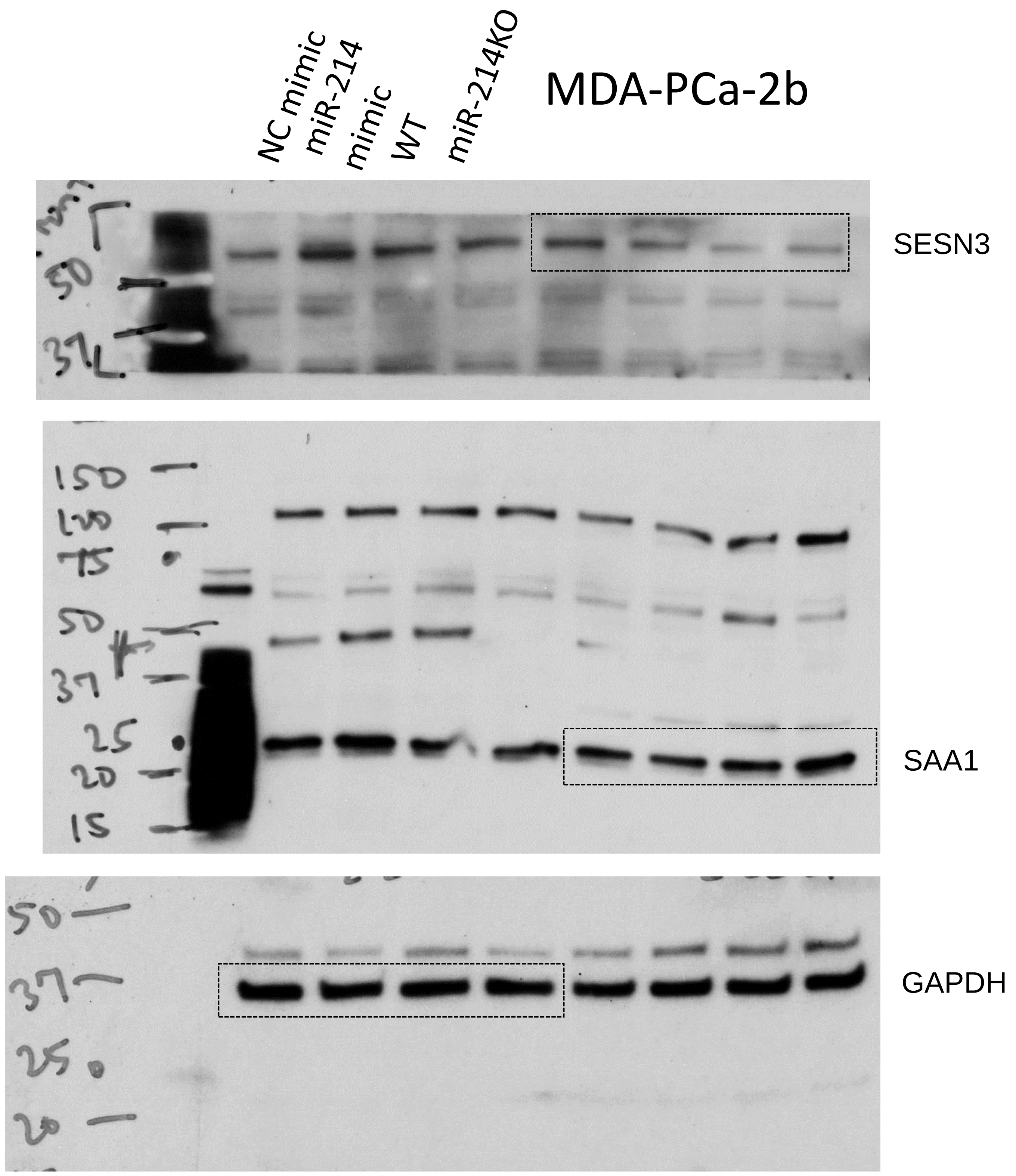

File S5.I (S5.I): Full length images of blots for Fig 5 I in main paper. Dotted rectangles indicate the regions used in the figures. PVDF membranes were used for probing with the respective primary antibody.

File S6.F

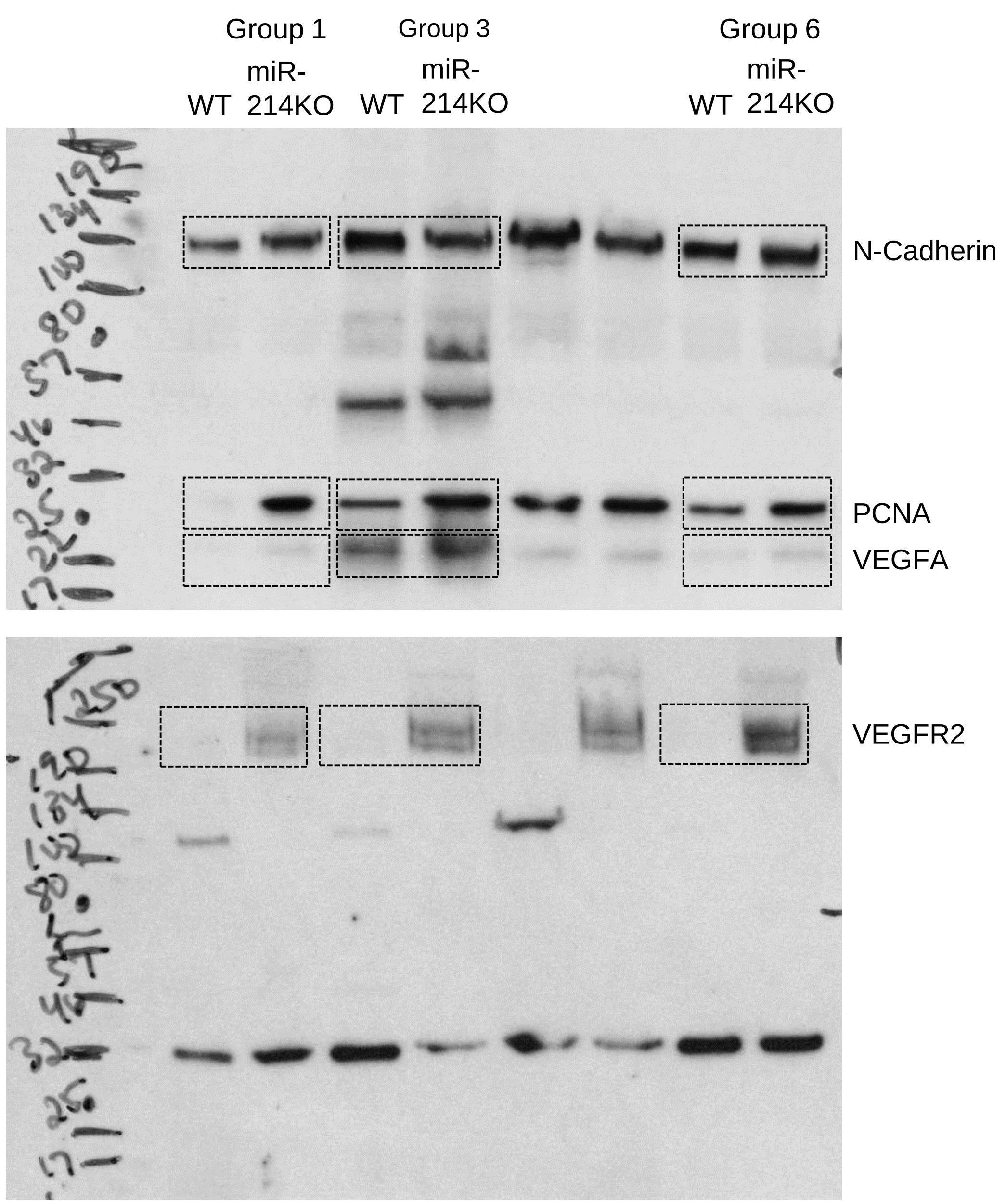

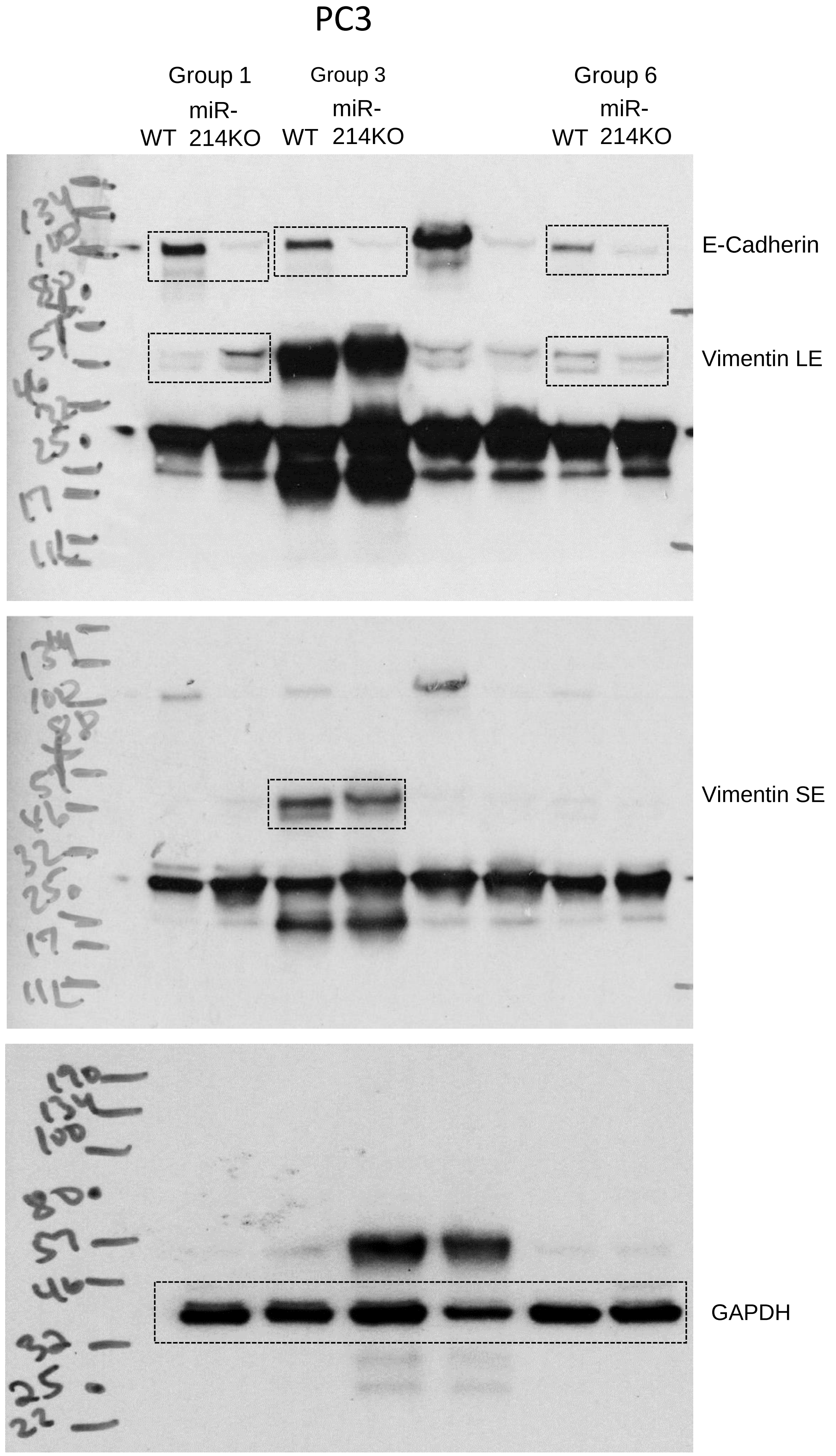

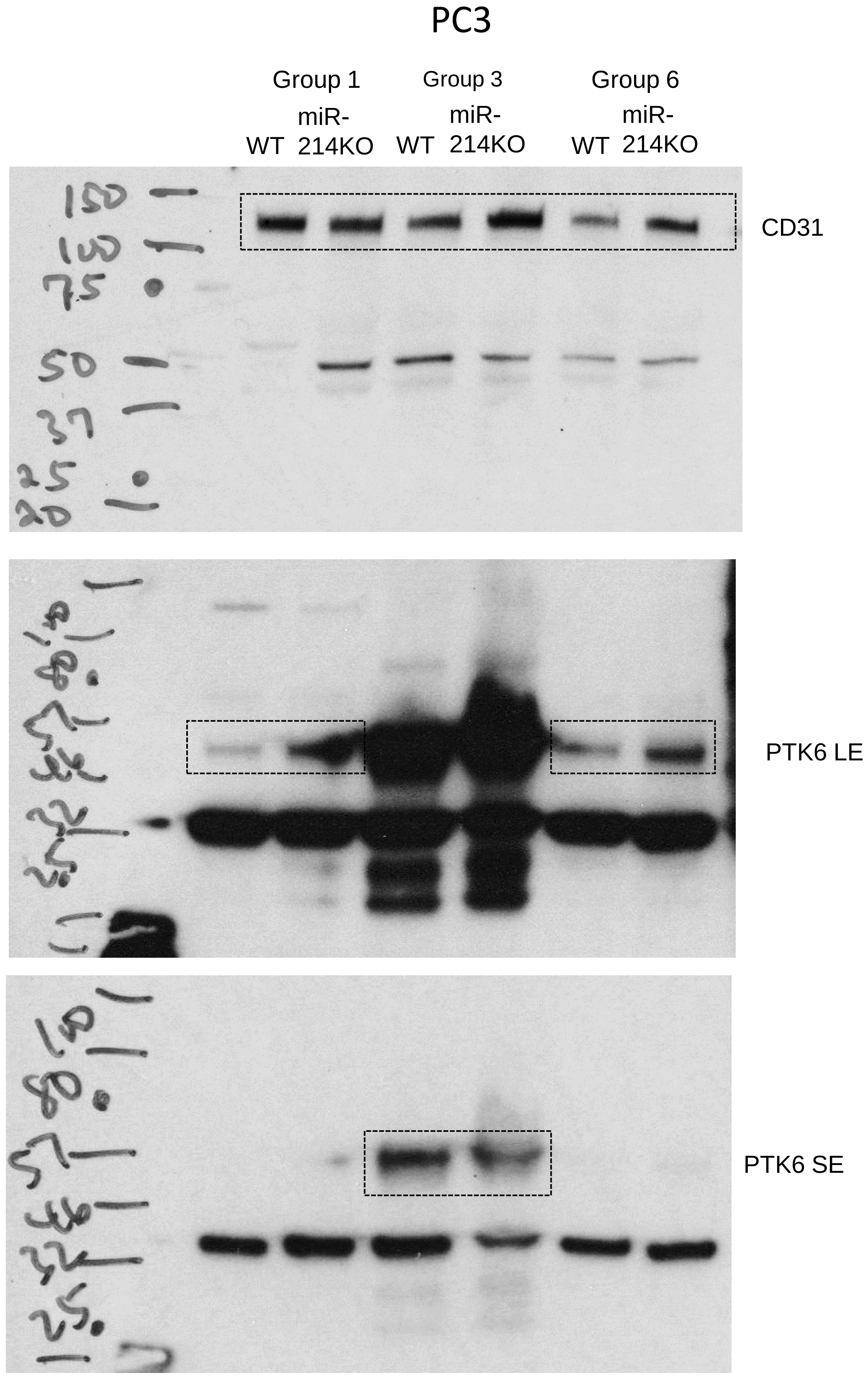

File S6.F (S6.F): Full length images of blots for Fig 6 F in main paper. Dotted rectangles indicate the regions used in the figures. PVDF membranes were used for probing with the respective primary antibody.

Supplementary Fig File S2.B

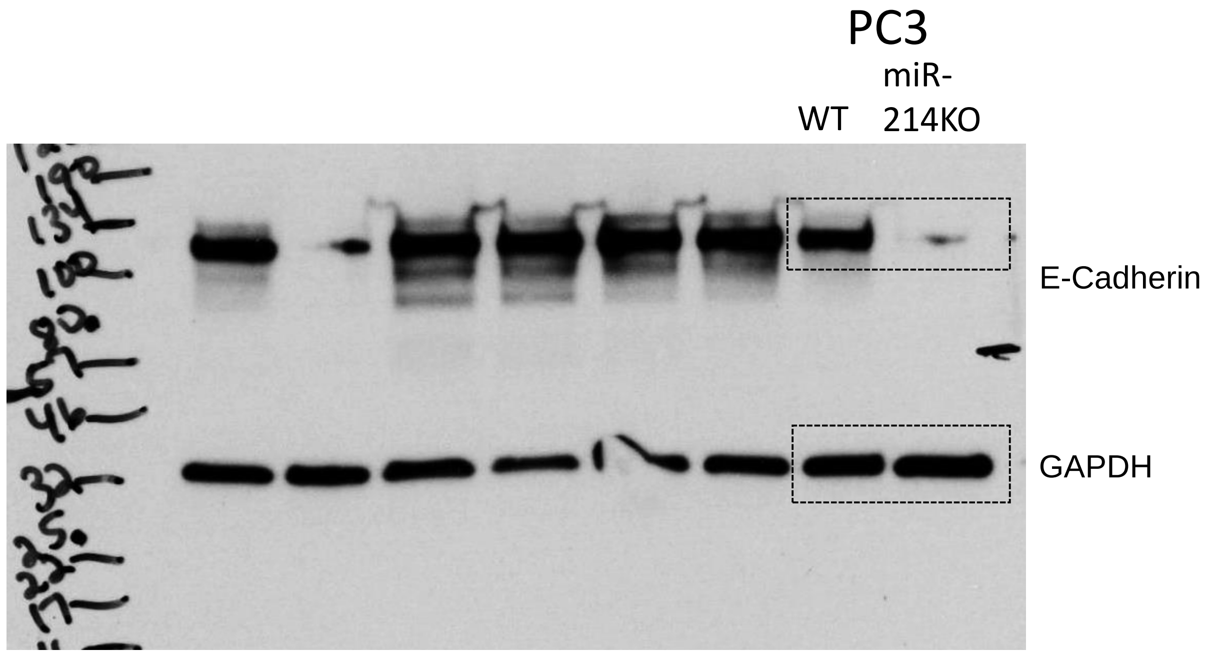

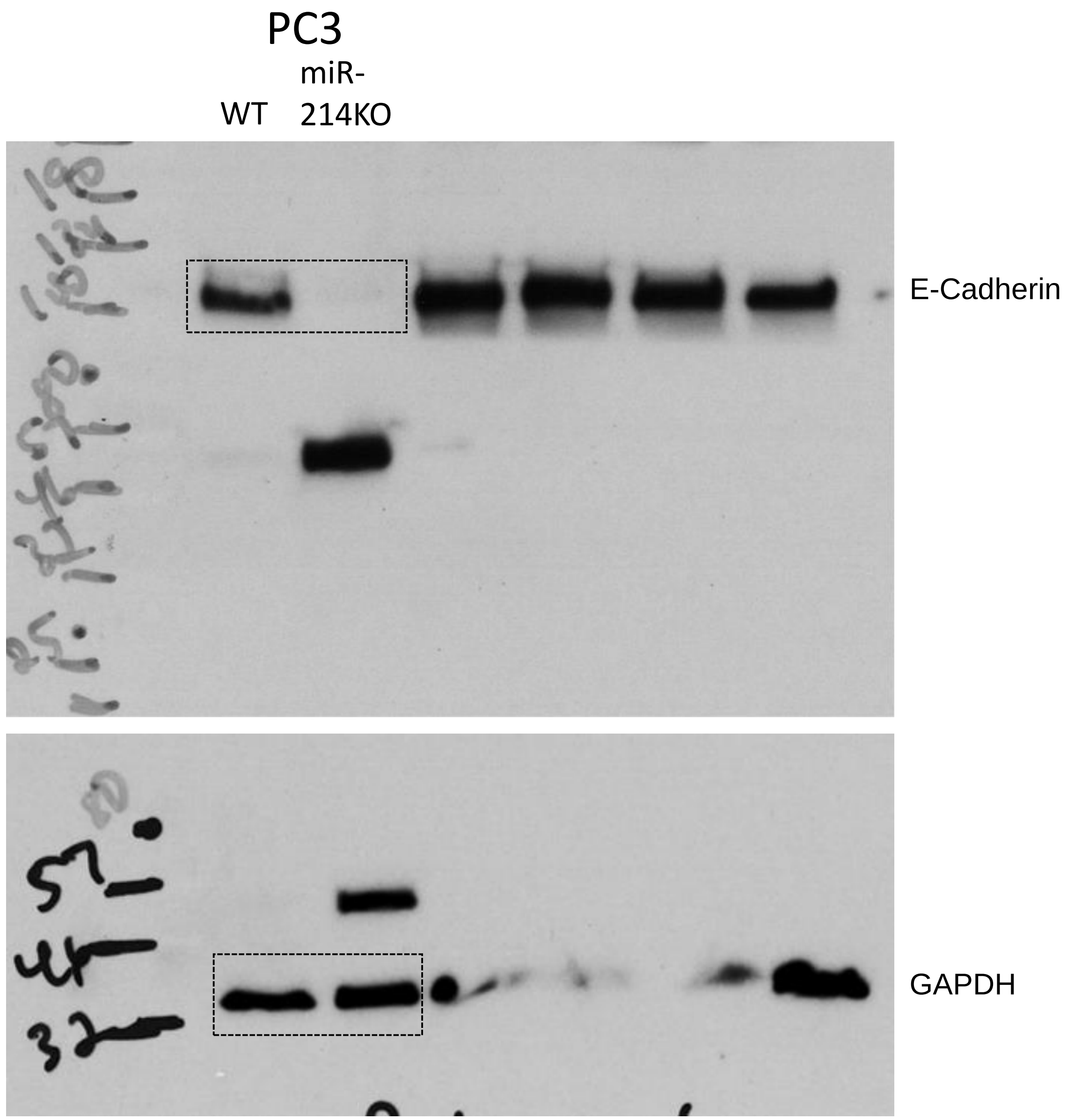

Supplementary Fig File S2.B (S2.B): Full length images of blots for Supp Fig S2 B in supplementary files. Dotted rectangles indicate the regions used in the figures. PVDF membranes were used for probing with the respective primary antibody.
