# Supplementary material for: Knockdown of microRNA-214-3p Promotes Tumor Growth and Epithelial-Mesenchymal Transition in Prostate Cancer
Source: Cancers (Basel). 2021 Nov 23;13(23):5875. doi: 10.3390/cancers13235875 (PMC8656576; doi:10.3390/cancers13235875)
Supplement: Supplementary file 1 [file cancers-13-05875-s001.zip › cancers-1450969-supplementary/Supplementary Final Figures v2 11-22-21.pdf]

Knockdown of microRNA-214-3p Promotes Tumor Growth and Epithelial-Mesenchymal Transition in Prostate Cancer

Patrice Cagle [1], Nikia Smith [1], Timothy O. Adekoya [1], Yahui Li [1], Susy Kim [2], Leslimar Rios-Colon [1], Gagan Deep [2,3], Suryakant Niture [1], Christopher Albanese [4], Simeng Suy [5], Sean P. Collins [5] and Deepak Kumar [1,6,*]

[1] Julius L. Chambers Biomedical Biotechnology Research Institute, North Carolina Central University, Durham, NC 27707, USA; pcagle@nccu.edu (P.C.); nsmith47@nccu.edu (N.S.); tadekoya@nccu.edu (T.O.A.); yli6@nccu.edu (Y.L.); lrioscolon@nccu.edu (L.R-C.); sniture@nccu.edu (S.N.)
[2] Department of Cancer Biology, Wake Forest School of Medicine, Winston-Salem, NC 27157, USA; sukim@wakehealth.edu (S.K.); gdeep@wakehealth.edu (G.D.)
[3] Wake Forest Baptist Comprehensive Cancer Center, Wake Forest School of Medicine, Winston-Salem, NC 27157, USA
[4] Lombardi Comprehensive Cancer Center, Department of Oncology, Georgetown University Medical Center, Washington, DC 20057, USA; Albanese@georgetown.edu
[5] Department of Radiation Medicine, Georgetown University Hospital, Washington, DC 20007, USA; suys@georgetown.edu (S.S.); spc9@georgetown.edu (S.P.C.)
[6] Department of Pharmaceutical Sciences, North Carolina Central University, Durham, NC 27707, USA
*Correspondence: dkumar@nccu.edu; Tel.: +1-919-530-7017

S1

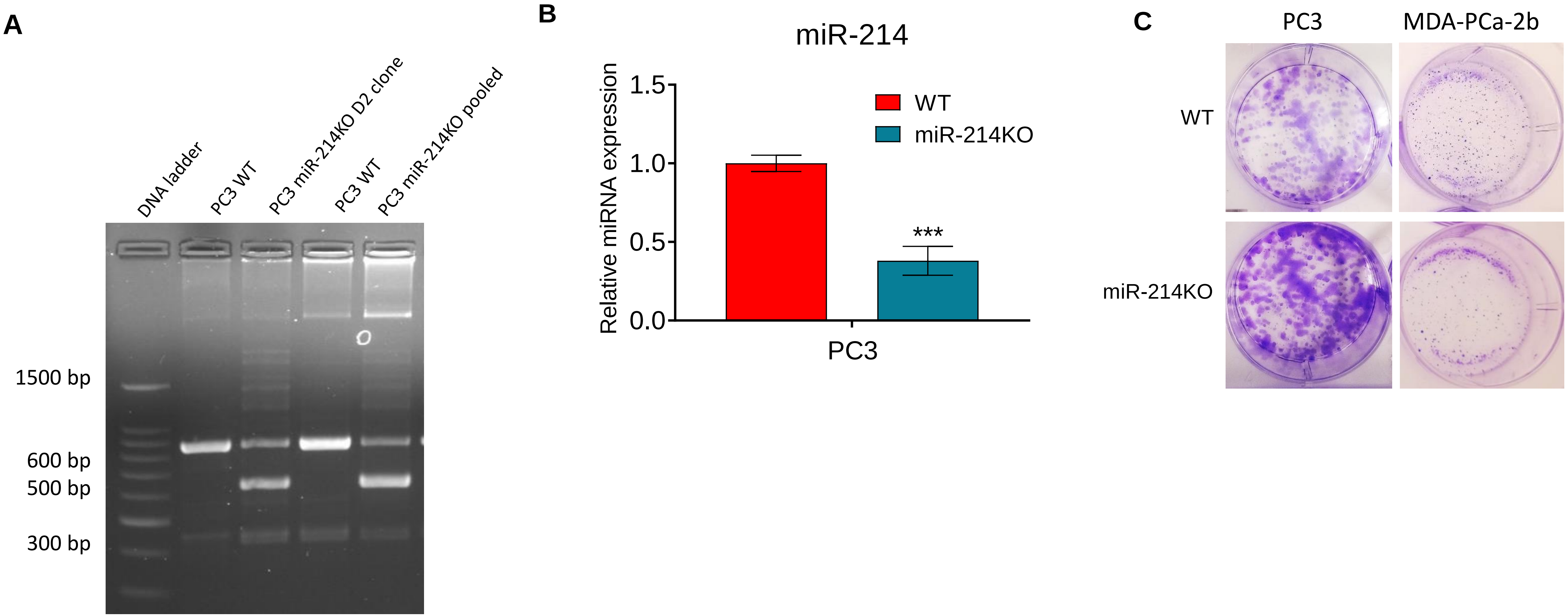

Supplementary Figure S1. Downregulation of miR-214 in PCa cell lines (A) DNA cleavage of miR-214 in PC3 clonal (D2 clone) and pooled cells by CRISPR/Cas9 is detected by PCR assay. (B) The relative expression of miR-214 was determined by qRT-PCR in WT PC3 cells compared to pooled miR-214KO PC3 cells. MiRNA levels were normalized to that of U44 RNA. Data are presented as mean ± SEM, *** $p < 0.0005$. (C) Representative images of the effect of knockdown of miR-214 on PC3 and MDA-PCa-2b cell colony formation were determined by colony formation assay.

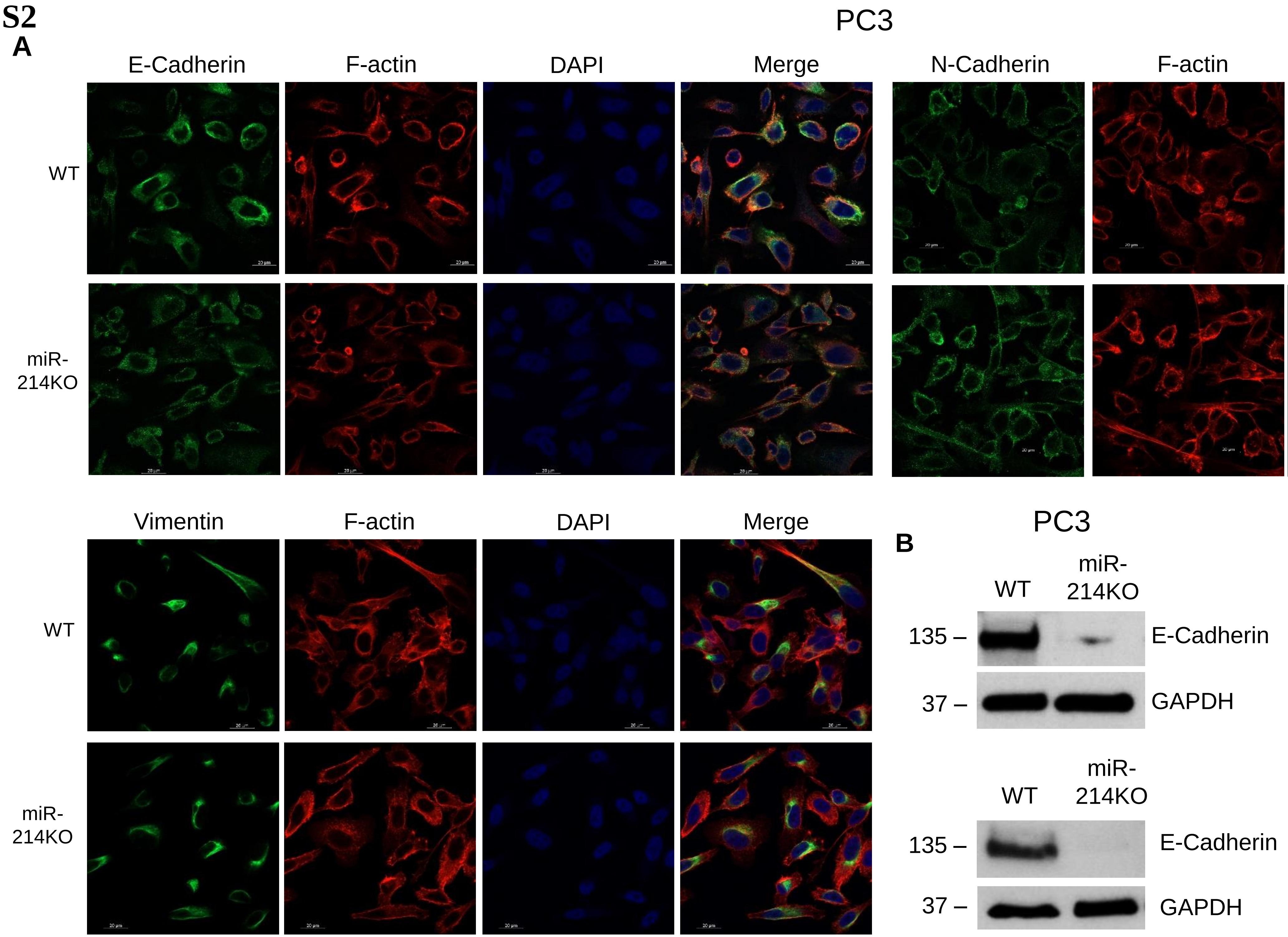

Supplementary Figure S2. Immunofluorescence staining of EMT markers and E-Cadherin protein expression in PC3 cells (A) F-actin, E-Cadherin, N-Cadherin, and Vimentin staining of miR-214 WT and KO PC3 cells. (B) Additional western blot analysis of E-Cadherin expression, from different PC3 cell lysates.

S3

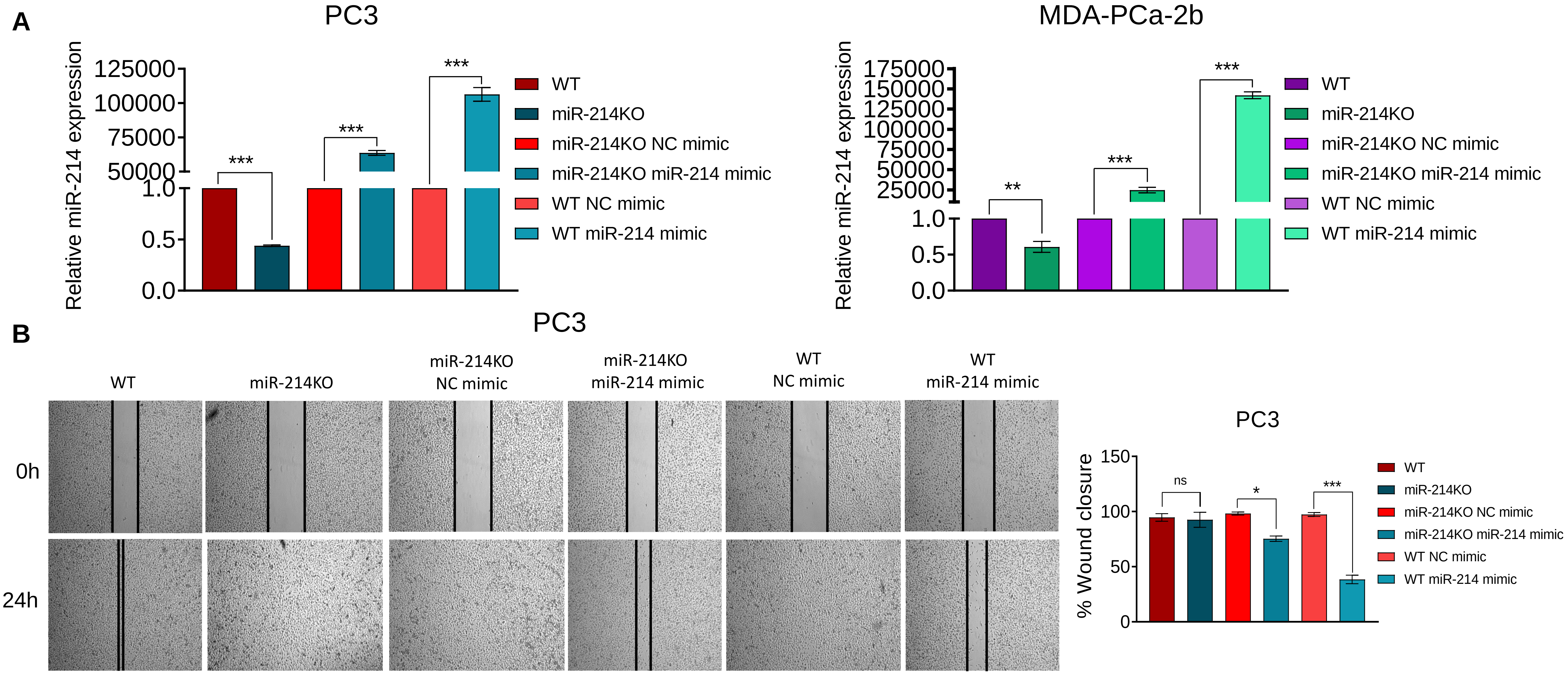

Supplementary Figure S3. Effect of miR-214 overexpression in miR-214-depleted PCa cells (A) Overexpression of miR-214 increased the expression of miR-214 in miR-214KO miR-214 mimic and WT miR-214 mimic PC3 and MDA-PCa-2b cells compared to miR-214KO NC mimic and WT NC mimic, respectively. The expression was determined by qRT-PCR, and U44 served as an internal control. (B) Wound healing assays were performed to investigate the changes of WT and miR-214KO PC3 cell migration ability upon overexpression of miR-214 compared with their negative controls. Representative photographs are presented in the left panels, the scratch area was calculated and graphically shown in the right panel. Data are presented as mean ± SEM one-way ANOVA using Tukey's multiple comparisons test, *$p < 0.05$, ***$p < 0.0005$, NS = not significant.

Table S1. Primer sets used for qRT-PCR

Primer sets	Primers	Sequences (5′–3′)
PTK6	Forward	CCTCTCCCATGACCACAATATC
	Reverse	GAGAATCCCAAAGGACCAGAC
E-Cadherin	Forward	CTC GAC ACC CGA TTC AAA GT
	Reverse	CCA GGC GTA GAC CAA GAA AT
N-Cadherin	Forward	GAC AGT TCC TGA GGG ATC AAA
	Reverse	CGA TTC TGT ACC TCA ACA TCC C
Vimentin	Forward	CAG CTT TCA AGT GCC TTT CTG
	Reverse	CTT GTA GGA GTG TCG GTT GTT
CXCR4	Forward	CCA CCA TCT ACT CCA TCA TCT TC
	Reverse	ACT TGT CCG TCA TGC TTC TC
PD-L1	Forward	TCA CTA CAC AGC CCT CCT AA
	Reverse	ACA CCA GAA TAT GGC CAA GAG
SESN3	Forward	CTT CAG CAC CGT ACA CCT AAT C
	Reverse	GAG GAA GAA GCA GGC AGA AA
ALK	Forward	CCC ATC TTT GAC CCT ACA GTT C
	Reverse	GAG TTC TGG TAG GCG TTG TT
SEMA6D	Forward	GTC CTC ATC ACC TGT GTC TTT
	Reverse	GGC GGA CTC TGC ATC TTT AT
PTN	Forward	CCA TGA AGA CCC AGA GAT GTA AG
	Reverse	CAG GCC TGG AAC TGG TAT TT
EPHA5	Forward	CCA GTG ATG TCT GGA GTT ATG G
	Reverse	GCC TTC CTC TAC CGC TTT AAT
HSD17B2	Forward	AGC ACT TTC TTC TCG GAC AC
	Reverse	GCC CTG AGC TCT TCT TGT ATT
CYP1B1	Forward	CTG TCT TGG GCT ACC ACA TT
	Reverse	GGA TCA AAG TTC TCC GGG TTA
ADORA1	Forward	ACC TGG AGG TCT TCT ACC TAA TC
	Reverse	TCT TCA GCT CCT TCC CAT AGT
SAA1	Forward	TGA TCA GGC TGC CAA TGA A
	Reverse	TCC TGA GAG CAG AGT GAA GA
DTX4	Forward	GTC CCT CAT CTC CCT CAT CTA T
	Reverse	GCA GAT TGG ACC AGA CCA TAT T
GAPDH	Forward	GGT GTG AAC CAT GAG AAG TAT GA
	Reverse	GAG TCC TTC CAC GAT ACC AAA G

Table S2. Top 50 dysregulated mRNAs in PC3 cells

Gene name	log2Fold Change	pvalue	padj	Gene name	log2Fold Change	pvalue	padj
Up-regulated				***Down-regulated***			
AL513323.1	9.93	6.94E-17	5.45E-16	KRT16	-9.50	1.61E-14	1.10E-13
GABRG3	8.95	2.99E-18	2.52E-17	OVOL1	-8.78	1.47E-13	9.35E-13
TCN1	7.74	0	0	TMPRSS13	-8.58	8.65E-13	5.21E-12
PLD5	7.52	4.46E-13	2.74E-12	MIR205HG	-8.53	7.74E-13	4.68E-12
ZFPM2	6.94	2.98E-124	2.24E-122	KRT4	-8.45	2.14E-12	1.25E-11
FO538757.2	6.87	1.53E-34	2.49E-33	AC006372.2	-8.45	2.26E-12	1.32E-11
PCSK5	6.83	7.16E-48	1.69E-46	CEACAM5	-8.37	2.50E-12	1.46E-11
AUTS2	6.37	1.98E-21	1.96E-20	KRT17	-8.25	4.72E-12	2.71E-11
TMEM108-AS1	6.33	3.34E-07	1.28E-06	DAPP1	-8.18	1.95E-15	1.41E-14
ADAMTS9	6.24	1.39E-20	1.32E-19	ZNF486	-7.84	1.28E-10	6.61E-10
NLGN4X	6.14	1.01E-30	1.43E-29	CASP14	-7.84	1.43E-10	7.37E-10
UBE2QL1	6.00	1.26E-135	1.05E-133	ZBTB7C	-7.80	7.65E-11	4.03E-10
CSMD2	5.89	4.92E-15	3.47E-14	MAL	-7.79	1.72E-10	8.80E-10
PDPN	5.56	5.89E-16	4.42E-15	SLC43A3	-7.73	8.91E-147	8.53E-145
PTPN5	5.48	7.77E-10	3.81E-09	MACC1	-7.72	3.48E-74	1.35E-72
SOBP	5.38	3.19E-20	2.99E-19	CALB2	-7.64	3.81E-10	1.91E-09
CNTN1	5.15	1.04E-41	2.09E-40	TRIM29	-7.40	2.29E-18	1.94E-17
MN1	4.79	1.47E-96	7.75E-95	AC245041.1	-7.37	9.43E-10	4.59E-09
RNF152	4.71	2.44E-18	2.06E-17	KCNK5	-7.35	2.40E-09	1.13E-08
RUBCNL	4.69	4.72E-09	2.17E-08	UGT1A1	-7.35	2.50E-09	1.17E-08
TMEM200A	4.68	5.92E-18	4.89E-17	WNT10A	-7.24	2.17E-09	1.02E-08
ESM1	4.68	0	0	CYP1B1	-7.17	4.27E-125	3.25E-123
GABRQ	4.59	2.30E-10	1.17E-09	ST14	-7.17	1.21E-291	3.85E-289
CHRDL1	4.51	1.71E-16	1.32E-15	FER1L6	-7.14	4.89E-17	3.87E-16
MUM1L1	4.48	7.94E-15	5.52E-14	S100A14	-7.06	4.47E-227	8.45E-225

Table S3. Top 50 dysregulated mRNAs in MDA-PCa-2b cells

Gene.name	log2Fold Change	pvalue	padj	Gene.name	log2Fold Change	pvalue	padj
Up-regulated				***Down-regulated***			
MUC12	7.81	1.64E-10	7.04E-10	MYCN	-7.17	1.10E-08	4.12E-08
ZNF486	7.47	1.34E-09	5.39E-09	AUTS2	-7.00	6.72E-36	8.49E-35
FRG2HP	7.38	2.21E-09	8.72E-09	CPA6	-6.97	3.38E-08	1.22E-07
PI15	6.74	0	0	NKX2-1	-6.58	1.54E-198	2.08E-196
EPHA6	6.25	4.90E-13	2.50E-12	WIF1	-6.43	1.17E-81	3.92E-80
AC021534.1	6.08	1.11E-06	3.51E-06	SFTA3	-6.42	1.32E-09	5.29E-09
MRAP2	5.43	9.46E-10	3.83E-09	GSTA2	-6.10	1.21E-26	1.14E-25
LINC01518	5.42	6.60E-07	2.13E-06	NKX2-8	-5.85	1.07E-97	4.61E-96
TRIM49	5.40	6.98E-07	2.25E-06	POU6F2	-5.66	1.54E-10	6.65E-10
CNTN5	5.26	7.81E-243	1.69E-240	PCSK5	-5.44	5.24E-15	3.02E-14
GPC5-AS1	5.22	3.72E-85	1.33E-83	HOTAIR	-5.23	8.80E-41	1.28E-39
CREB5	5.18	2.48E-06	7.53E-06	GBA3	-4.72	4.18E-09	1.62E-08
MBNL2	5.13	5.29E-23	4.37E-22	ADORA1	-4.64	1.57E-37	2.07E-36
DCAF4L2	5.09	1.32E-152	1.17E-150	TMTC1	-4.52	0	0
LRP12	5.08	1.09E-51	2.05E-50	GSTA1	-4.46	5.77E-210	8.77E-208
AP005435.1	5.08	2.10E-22	1.69E-21	GLB1L3	-4.42	2.85E-14	1.57E-13
STMN4	5.07	4.61E-13	2.36E-12	RORB	-4.36	4.74E-22	3.76E-21
PCGEM1	4.77	1.27E-30	1.38E-29	JCAD	-4.34	3.55E-95	1.46E-93
AP001610.2	4.72	2.86E-07	9.56E-07	HSD17B2	-4.30	1.96E-10	8.38E-10
CORIN	4.70	7.22E-50	1.29E-48	LINC02463	-4.30	8.54E-09	3.23E-08
AC010967.1	4.69	3.29E-07	1.09E-06	HOXC12	-4.22	3.21E-123	1.99E-121
CHRM3	4.64	2.65E-270	6.86E-268	EML6	-4.12	5.60E-200	7.77E-198
DYNC1I1	4.63	7.12E-29	7.22E-28	HPSE2	-4.09	2.49E-275	6.69E-273
PLPPR4	4.61	3.69E-16	2.24E-15	CHN2	-4.09	3.08E-09	1.20E-08
CA9	4.53	2.17E-12	1.07E-11	C4orf19	-4.05	6.27E-45	9.95E-44
